# Traceability, reproducibility and wiki-exploration for “à-la-carte” reconstructions of genome-scale metabolic models

**DOI:** 10.1371/journal.pcbi.1006146

**Published:** 2018-05-23

**Authors:** Méziane Aite, Marie Chevallier, Clémence Frioux, Camille Trottier, Jeanne Got, María Paz Cortés, Sebastián N. Mendoza, Grégory Carrier, Olivier Dameron, Nicolas Guillaudeux, Mauricio Latorre, Nicolás Loira, Gabriel V. Markov, Alejandro Maass, Anne Siegel

**Affiliations:** 1 IRISA, Univ Rennes, Inria, CNRS, Rennes, France; 2 ECOBIO, Univ Rennes, CNRS, Rennes, France; 3 UMR 6004 ComBi, Université de Nantes, CNRS, Nantes, France; 4 Centro de Modelamiento Matemático, Universidad de Chile, Santiago, Chile; 5 Facultad de Ingeniería y Ciencias, Universidad Adolfo Ibáñez, Santiago, Chile; 6 Centro para la Regulación del Genoma (Fondap 15090007), Universidad de Chile, Santiago, Chile; 7 Laboratoire de Physiologie et de Biotechnologie des Algues, IFREMER, Nantes, France; 8 Instituto de ciencias de la ingeniería, Universidad de O'Higgins, Rancagua, Chile; 9 Instituto de Nutrición y Tecnología de los Alimentos, Universidad de Chile, Santiago, Chile; 10 UMR 8227, Integrative Biology of Marine Models, Station biologique de Roscoff, Sorbonne Université, CNRS, Roscoff, France; Chalmers University of Technology, SWEDEN

## Abstract

Genome-scale metabolic models have become the tool of choice for the global analysis of microorganism metabolism, and their reconstruction has attained high standards of quality and reliability. Improvements in this area have been accompanied by the development of some major platforms and databases, and an explosion of individual bioinformatics methods. Consequently, many recent models result from “à la carte” pipelines, combining the use of platforms, individual tools and biological expertise to enhance the quality of the reconstruction. Although very useful, introducing heterogeneous tools, that hardly interact with each other, causes loss of traceability and reproducibility in the reconstruction process. This represents a real obstacle, especially when considering less studied species whose metabolic reconstruction can greatly benefit from the comparison to good quality models of related organisms. This work proposes an adaptable workspace, AuReMe, for sustainable reconstructions or improvements of genome-scale metabolic models involving personalized pipelines. At each step, relevant information related to the modifications brought to the model by a method is stored. This ensures that the process is reproducible and documented regardless of the combination of tools used. Additionally, the workspace establishes a way to browse metabolic models and their metadata through the automatic generation of ad-hoc local wikis dedicated to monitoring and facilitating the process of reconstruction. AuReMe supports exploration and semantic query based on RDF databases. We illustrate how this workspace allowed handling, in an integrated way, the metabolic reconstructions of non-model organisms such as an extremophile bacterium or eukaryote algae. Among relevant applications, the latter reconstruction led to putative evolutionary insights of a metabolic pathway.

## Author summary

Genome-scale metabolic models describe an organism’s metabolism. Building good models requires the integration of all relevant available information, obtained by exploring different data types and biological databases. This process is not straightforward and choices are made along the way, for example, which data is analyzed, with what tools. It matters that all reconstruction steps are well documented so that models can be fully exploited by the community. Having this metadata allows other researchers to reproduce, improve or reuse a model as a blueprint to create new ones. Sadly, this information is usually scattered and its proper distribution is the exception rather than the norm when using “à la carte” pipelines that combine main platforms and individual tools. We created a platform for “à la carte” metabolic model generation that responds to the need of transparency and data-connection in the field. It includes a battery of tools to exploit heterogeneous data through customizable pipelines. At each step, relevant information is stored, ensuring reproducibility and documentation of processes. Furthermore, exploration of models and metadata during the reconstruction process is facilitated through the automatic generation of local wikis. This view offers a user-friendly solution to iteratively explore genome-scale metabolic models produced with personalized pipelines and poorly interoperable tools. We highlight these benefits by building models for organisms with various input data. Among them, we show why the combination of heterogeneous information is necessary to elucidate specificities of *Tisochrysis lutea*, a eukaryotic microalga, for anti-oxidant production.

## Introduction

The emergence of technologies able to produce massive data in all omics sciences has raised new challenges to handle, connect, exploit and distribute such information. Genome-scale metabolic models (GSMs) represent a successful application of integration of various types of omics data. GSMs are structured knowledge bases describing a specific organism metabolism [1]. They are characterized by two main features. First, they describe an organism metabolic network by incorporating biochemical reactions at a genome scale and associating them to the corresponding enzymes and their coding genes. Second, through formal mathematical formulation they can exploit this knowledge and predict the state of the network in different growth scenarios [2]. Prediction of phenotypes has allowed GSMs to be applied for several purposes such as, guiding metabolic engineering efforts to achieve an increased production of target metabolites [3], identification of drug targets [4] and more recently, prediction of interactions in microbial communities [5].

To accomplish such applications successfully, an effort to correctly incorporate all the relevant available information must be made first, so that the quality of the reconstructed GSM is the best possible. To this end, a well-described protocol for generating high-quality GSMs has been made available [6]. Additionally, there are several databases such as KEGG [7], BioCyc [8], BiGG [9] or Model SEED [10] that aggregate metabolic data on which GSMs can be built [11,12]. For instance, the contents of the MetaCyc database have been curated from 54,000 articles [8]. Moreover, many independent methods have been developed to generate GSMs, mostly based on the aforementioned databases, including some toolboxes and workspaces. These latter allow a user to chain several tools into pipelines and have proven their efficiency in building high-quality GSMs. Among them are Pathway Tools [13], the Raven Toolbox [14] and The SEED [10]. Pathway Tools was the first platform connecting biological metadata by relying on the BioCyc database via their own internal format. It also ensures traceability and reproducibility for the tools implemented in the platform. The Raven Toolbox integrated multiple data source types to encompass the variability of available data in reconstruction processes. The SEED proposed a complete automatic generation of models for prokaryotes and plants. There are also online workspaces such as Galaxy [15] or KBase [16] that enable the creation and customization of pipelines, while exploiting their own intrinsic databases. Most of these platforms internally trace the source of every reaction and metabolite in a GSM, that we call process metadata. For instance, Pathway Tools supports the use of evidence codes, citations, and user comments to document the origin and reason why information is included in a model. This information allows reports to be produced for comparing different versions of a model provided that the model construction is completely built within a single platform.

Thiele and Palsson’s protocol steps are generally followed during reconstructions although customized to the availability of data sources and tools. As an example, the study of non-model organisms may involve comparisons to genomes and metabolisms of several taxonomically-related organisms. In such cases, the output of a main platform requires adjustments assisted by a choice of specialized tools. In this sense, as illustrated in Table 1 (see Results), many recent GSMs were obtained by combining a major platform with additional methods relying on several databases, leading to “à la carte” reconstruction pipelines. The output of such personalized pipelines is exported in standard formats such as SBML or stoichiometric matrices, leaving out information about sources of reactions (e.g., the method and reason why a reaction was added to a model) and process metadata which cannot be recovered with versioning systems.

**Table 1:** Survey of 19 GSM reconstruction procedures. Variability of reference ID-databases, available annotations and metadata about reactions and heterogeneity of the methods used in each GSM reconstruction pipeline.

Species			Ref. database		Annotation of reactions					Methods and templates for reconstruction					
Species	Group	Reference	Database used	Database crossreferences	Reactions	Unreferenced reactions	Literature or experimental justification	Confidence score	Gene association	Template model(s)	Orthology	Genome annotation	Gap-filling	Flux analysis	Manual curation
*Synechocystis sp*. *PCC6803*	*Cyanobacteria*	[27]	2	-	882	79	-	-	✓	3	-	✓	-	✓	✓
*Synechocystis sp*. *PCC6803*	*Cyanobacteria*	[28]	2	✓	1156	23	✓	-	✓	8	✓	✓	✓	✓	✓
*Synechocystis sp*. *PCC6803*	*Cyanobacteria*	[29]	1	✓	759	166	✓	✓	✓	9	✓	✓	-	✓	-
*A*. *ferrooxi-dans* ATCC 23270	*Proteobacteria*	[30]	3	-	615	4	✓	✓	✓	0	-	✓	-	✓	✓
Salmonella typhimurium LT2	*Proteobacteria*	[31]	2	-	2545	12	✓	✓	✓	2	-	-	-	✓	✓
*Leptospira* (4 strains)	*Spirochaetes*	[32]	2	✓	1017	52	-	-	NA	0	✓	✓	✓	✓	-
*Staphylococcus aureus* (64 strains)	*Firmicutes*	[24]	4	✓	1507	17	-	-	✓	15	✓	✓	✓	✓	✓
*Enterococcus faecalis V583*	*Firmicutes*	[33]	1	-	706	355	-	✓	✓	4	✓	-	-	✓	✓
*Clostridium ljungdahlii*	*Firmicutes*	[34]	3	✓	785	0	-	-	✓	4	✓	✓	✓	✓	✓
*Lactobacillus planta-rum* WCFS1	*Firmicutes*	[35]	1	-	761	371	✓	-	✓	0	✓	✓	-	✓	✓
*Methanosarcina acetivorans*	*Euryarchaeotes*	[36]	3	-	845	180	-	-	✓	2	-	✓	-	✓	✓
*Pichia pastoris* GS115	*Ascomycetes*	[37]	1	-	1202	NA	✓	-	✓	1	✓	-	✓	✓	✓
*Saccharomyces cerevisiae*	*Ascomycetes*	[38]	1	-	1412	NA	-	-	✓	2	-	✓	✓	✓	✓
*Chlamydomonas reinhardtii*	Green algae	[39]	2	✓	2190	632	✓	-	✓	2	✓	-	-	✓	✓
*Chlamydomonas reinhardtii*	Green algae	[40]	2	✓	2394	689	✓	✓	✓	1	✓	-	✓	✓	✓
*Ectocarpus siliculosus*	Brown algae	[41]	2	✓	1866	52	-	✓	✓	1	✓	✓	✓	✓	✓
*Arabidopsis thaliana*	Eudicots	[42]	2	-	1567	0	✓	-	✓	0	-	✓	✓	✓	✓
*Zea mays*	Monocots	[43]	2	-	8525	27	-	-	✓	1	✓	✓	✓	✓	✓
*Homo sapiens*	Primates	[44]	1	-	3742	20	✓	✓	✓	0	-	-	✓	✓	✓

The reproducibility of these personalized reconstructions is threatened by the lack of metadata availability caused by the use of multiple methods and toolboxes in “à la carte” pipelines. Hence, tracking of process metadata is needed when using tools that accomplish dedicated subtasks of metabolic model reconstruction without being part of an existing platform. This would allow transparency throughout the reconstruction process, as discussed by Heavner and Price [17].

To circumvent these issues, we present the workspace *AuReMe* (AUtomatic REconstruction of MEtabolic models). *AuReMe* is designed to house “à la carte” reconstructions and analysis of GSMs while ensuring that their metadata is properly stored and can be efficiently explored and distributed. In particular, it can complement reconstructions provided by external platforms, such as Pathway Tools for which the *AuReMe* import preserves the existing process metadata. The *AuReMe* workspace encompasses tools (e.g. Cobrapy [18], PSAMM [19], OrthoMCL [20], Inparanoid [21], Pantograph [22] and its own internal tools: MeneTools, PADMet-utils) useful for essential steps in GSM reconstruction (import of annotation-based networks, template-based orthology predictions, gap-filling, manual curation). GSM analysis during the manual curation can be undergone with both flux-based and graph-based criteria. These tools can be connected through customized pipelines suited for diverse user needs, and offer different levels of flexibility in terms of supported input data, used tools and their interconnection as well as the possibility to perform manual intervention at different steps of the process. The customized pipeline can be safely run and reproduced thanks to log files which describe the exact chaining of tools used within the pipeline together with their parameters.

Data management using the newly developed *PADMet* python package allows storing information about which method was used to include a reaction in the model (process metadata) together with classical information about reactions, compounds and pathways attributes (the so-called biological metadata). All of them, along with the model itself, can be automatically integrated in a local wiki interface dedicated to monitoring and facilitating the reconstruction process. By structuring and linking data (methods used in pipelines, reactions, compounds, pathways, genes, etc.), and by integrating semantic search functionalities, this view offers a user friendly solution to iteratively explore GSM produced with personalized pipelines and poorly interoperable tools. The generation is made locally to assist the user during the reconstruction. GSM updates are made through the use of assisted manual curation forms rather than by wiki edition for the sake of traceability. Once the model is fully reconstructed, it can be shared either with the SBML files, through online deployment of the generated wiki, or integrated in other platforms via several output formats provided.

From a technical point of view, *AuReMe* can be viewed as a workflow controller allowing GSM (possibly initiated with a major platform) to be customized with “à la carte” pipelines of dedicated tools while keeping a record of the methods used. This ensures the reproducibility of the GSM customization procedure. The workflow controller, based on the Docker technology, is associated with a local data manager (*PADMet* python package). It monitors and facilitates the ongoing reconstruction via a wiki, which is a view of the model linked metadata and is automatically generated with the MediaWiki technology.

We illustrate the benefit of our approach on several case-studies. Among them, we show why the combination of heterogeneous information is absolutely necessary to elucidate the specificities of *Tisochrysis lutea*, a eukaryotic microalga currently used in oyster farming and studied in the context of bio-fuel applications. Its metabolic network was reconstructed by relying both on annotations and orthologies with four different template metabolic networks. This analysis strongly suggests that *T*. *lutea* has the same capability as *Chlamydomonas reinhardtii* to produce carnosine, a specific antioxidant dipeptide consisting of beta-alanine and L-histidine. Beta-alanine is produced through two distinct pathways, including one initiated by aspartate. On the contrary, only one of them was identified in the macroalga *Ectocarpus siliculosus*. Interestingly, the missing pathway producing beta-alanine from aspartate was identified in a symbiont of its algal wall: *Candidatus* Phaeomarinobacter ectocarpi [23], paving the way to the study of organisms communities at the metabolic level.

## Results

### Heterogeneity of reconstruction processes reveals heterogeneity in traceability and metadata availability

We surveyed the reconstruction procedures of 19 published GSMs listed in Table 1. These GSMs were selected because they cover main phylogenetic branches including eukaryotes (ascomycete yeasts, green and brown algae, terrestrial plants and human), eubacteria (cyanobacteria, proteobacteria, spirochaetes and firmicutes) and euryarcheote archea. GSMs were selected to include highly studied organisms such as *Saccharomyces cerevisiae* and non-model species as well, such as *Acidithiobacillus ferrooxidans*. Thus, we avoid bias related to the level of information available for the reconstructed organisms or its phylogenetical cluster.

We compared these GSMs in terms of metabolic model content, selected databases, available metadata and reconstruction processes. We first observed that most models display “biological” metadata, i.e., metadata related to the model itself (gene associations, external references, etc.) and to its connection to other resources of knowledge (template metabolic networks, protein or chemical databases, etc.). This information is currently provided in SBML files, the most widespread export format. Then we compared these GSMs in terms of selected databases and reconstruction processes.

We observed that annotation, use of one or several (up to 15) template models, gap-filling and manual curation are four widely shared steps, which are consistent with the general methodology described by Thiele and Palsson [6]. However, we also noticed that different tools and methods were used in the reconstruction, which confirms the hypothesis of “à la carte” reconstruction pipelines. In addition, we noted that several databases of reactions were usually used for reconstructing models (presence of identifiers related to the main databases: KEGG, BiGG, BioCyc, Model SEED). In fact, one database and one method of reconstruction is rarely enough to obtain a model. For example, Bosi et al [24], included information from all the aforementioned databases to reconstruct 64 models of *Staphylococcus aureus*. Notice however that use of multiple reaction database requires a consolidated curation procedure to avoid duplicate reactions [25].

Starting from these observations, we investigated further the possibility of tracing the origin of reactions. This is a main part of what we define as “process” metadata (Supp. Fig A in S1 File), related to the reconstruction processes: steps at which reactions were added (automatic reconstruction, gap-filling, manual curation), and the information sources they depend on (annotation, orthology, etc.). These metadata make it possible to i) locate and connect the studied model along with other models and knowledge resources and ii) trace the reconstruction processes and ensure their reproducibility. Our study of the 19 GSMs highlighted that, when available, the process metadata of reactions were provided on multiple supports that were often neither machine-readable (pdf files, Excel files, notes in SBML files) nor suitable for further exploration. There was often no means to decipher at which step of the reconstruction and the reason why a particular reaction was added, making reproducibility of the model generation more difficult. In particular, manual curation was not always explicit. The only evidence that this process had been conducted was the presence of reactions with unreferenced identifiers, which do not match identifiers in the database(s) described as being used for the reconstruction process [26].

We concluded that missing metadata, particularly the process-related ones, is mainly attributed to the unstandardized and unrecorded passing through multiple tools used during model reconstruction. This causes the lack of traceability of reactions origin when studying the output models Thus, this survey advocates the need for tracking and storing metadata and for ways to explore and/or distribute these metadata along with the GSM.

### A package and a workspace to personalize and trace GSM reconstruction

**Fig 1:**
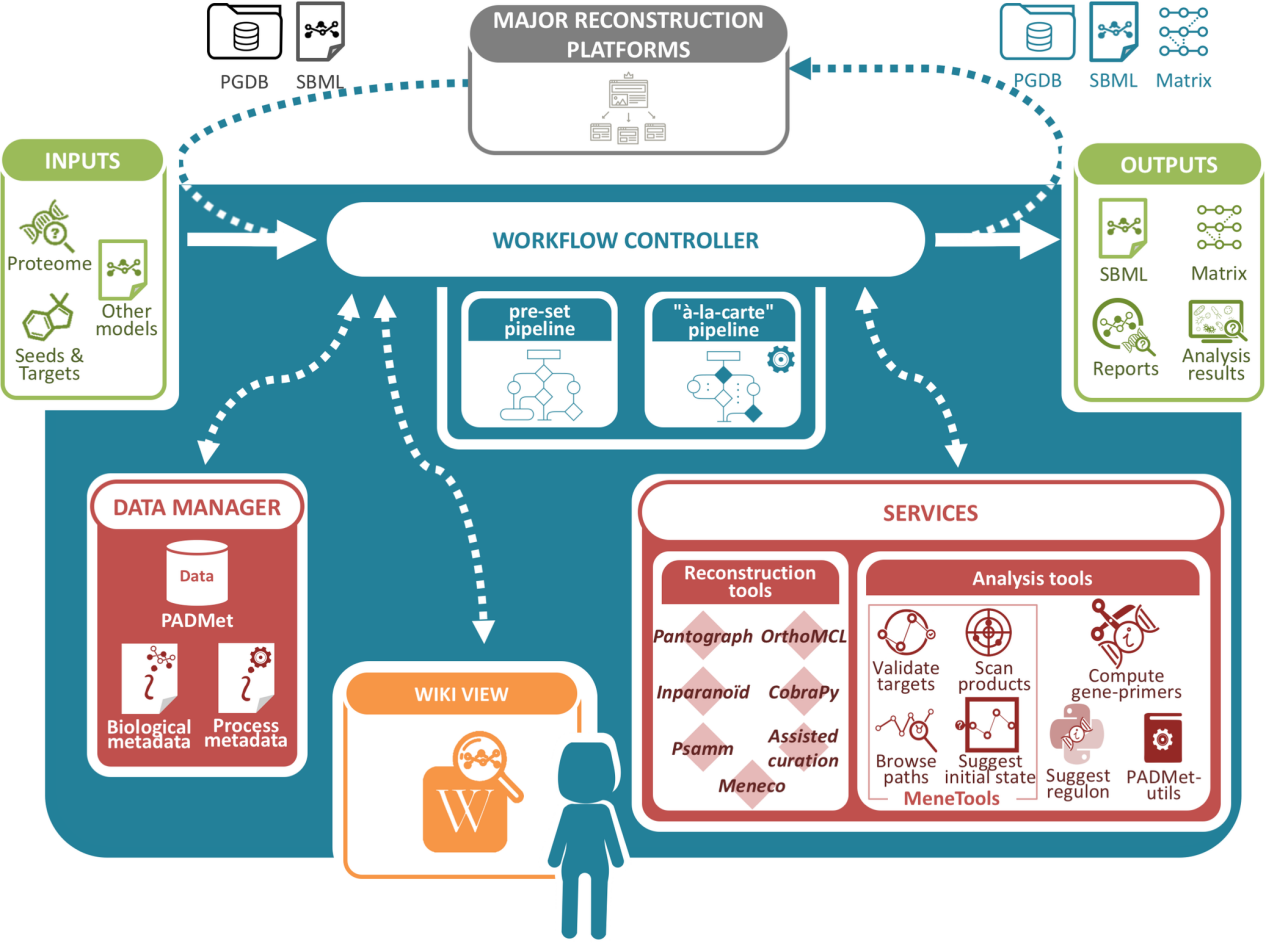
AuReMe workspace. *Overview of the AuReMe workspace*. Admissible inputs include standard formats in genomics and metabolic model fields that can be outputs of major reconstruction platforms. *AuReMe* acts as a workflow controller to administer the reconstruction or modification of the GSM performed by heterogeneous and independent tools. The latter are part of the services of *AuReMe* (reconstruction tools, analyses, manual curation) and can be chained together, either in a pre-set pipeline or in a customized one. In any case the *PADMet* data manager stores adequate information regarding the model and its metadata, most importantly the process ones, that keeps track of the modifications performed (at what step a reaction was added, by which tool etc.). At any time, the reconstruction can be monitored locally via an automatically-generated wiki that informs the user about the state of the model. Outputs of *AuReMe* can be self-sufficient or be integrated again in many existing platforms.

As described previously, current methods allow the reconstruction of high-quality GSMs but do not always take into account the need for metadata storage and exploitation to facilitate the study and reproducibility of models. We designed a unified workspace *AuReMe* (AUtomated REconstruction of MEtabolic models) to house the “à la carte” reconstruction of GSMs (Fig 1). This workflow controller, based on the Docker technology is the conductor handling the order of methods used in personalized pipelines. It is associated with a local data manager, the Python package *PADMet* (Python library for hAndling metaData of METabolism), whose role is to store information related to the sequence of tools used in the pipeline. Finally, PADMet-utils encompasses several tools and methods to curate, analyze a GSM considering topological or flux modeling, generate wikis, produce reports and export the models.

*AuReMe* gathers academic-free tools and enables the design of reconstruction pipelines that are flexible and can suit various available data sources (genome annotation, template GSMs, protein sequences, etc.) while storing metadata to ensure reproducibility of reconstructions (Supp. Fig A in S1 File). It can follow four major steps of reconstruction processes: annotation or orthology-based modelings, gap-filling and manual curation. In addition, *AuReMe* supports most processes of the Thiele and Palsson protocol [6] by proposing tools and methods that facilitate analysis and storing of the results at each step related to experiments or exploration of literature. In particular, the refinements to reconstruction are strongly related to the management of metadata performed in *AuReMe*. Manual curation is assisted and formalized within forms to be filled before being integrated in the pipeline treatment. Additionally, analysis tools based on flux or topology are also included in the workspace. Contrary to existing platforms, *AuReMe* works with three major databases that are freely or academic-freely available: BiGG, ModelSEED and MetaCyc, for which some versions are already included. *AuReMe* also works with the KEGG database provided that the user has the appropriate licence. The use of those databases facilitates the open-data initiative, that our workspace wants to promote. At any time during the reconstruction process, the visualization of model data and associated metadata is available through the generation of a local wiki, that can be connected to the MetaCyc, the BiGG or theSEED database used for reconstruction. Each run of *AuReMe* requires to select a main reference database but reactions from other databases (such as those predicted with orthology-based methods from models using alternative database identifiers) can be inserted in the reference database after a mapping operation based on the MetaNetX dictionary [45].

The first feature of the *AuReMe* workspace is its adaptability to various input data and databases. The *PADMet* package format ensures the interoperability of knowledge, tools and data (Fig 1). A wide range of input data types and the three pre-set databases (see S1 File for details) enable the exploitation of all genomic and metabolic exploration within the workspace.

The second feature of the *AuReMe* workspace is the customization of a pre-set pipeline. For example, the result of an annotation-based reconstruction can be imported into the workspace with the purpose of being merged with one or several orthology-based network(s), or other pre-existing models. Gap-filling and topological or flux analysis can then be performed. All of these steps can be personalized through the pipeline creation (see S1 File). The *PADMet* data manager stores all the necessary information about the methods used to add reactions to the final network. It also stores in a log file how tools are chained and parameterized in order to allow the automatic reproduction of the reconstruction process. Metabolic model version tracking can be done by using a network comparison command line which reports all the differences (genes, reactions, compounds and pathways) between several GSMs, including several versions of a model.

The third feature of the *AuReMe* workspace is the possibility to reuse assisted and tracked manual curation in further versions of a GSM. These modifications to the model for including expert knowledge of biologists as well as ad-hoc literature are needed to enrich the quality of a model reconstruction. As done in KBase and Pathway Tools for instance, manual curation (creation, modification and deletion of metabolites/reactions) is assisted via the use of forms. All manual update operations are stored internally by the *PADMet* data-manager. This allows a user both to trace the reasons for adding the reactions and to automatically include ongoing manual curations in a future version of the model. The purpose is to ensure the consistency and sustainability of metadata, especially when the pipeline has to be run again, due to the availability of a better version of input data (new genome assembly for instance), of updated version of databases or of new version of tools included in the customized pipelines.

The last feature of the *AuReMe* workspace is to be opened and complementary to other platforms in order to facilitate further analyses. Connections can be made to use external analysis and reconstruction tools (Fig 1). Exports to SBML [46] (|v|3 format by default, |v|2 if needed), including biological metadata and some process metadata, or stoichiometric matrix formats suit most tools that work with metabolic models. Exported models created within the workspace can then be used in Cobra Toolbox [47], Raven toolbox [14], Cytoscape [48], etc. When a model is obtained by enriching an initial model produced with Pathway Tools (respectively, Kbase), the final model can be imported back in Pathway Tools (respectively, Kbase), in order to undergo further analysis and publication in BioCyc. For example, the *E*. *siliculosus* GSM presented in the results was initiated with a PGDB produced by Pathway Tools (1661 reactions), then enriched with 440 reactions using orthology, topological gap-filling and manual curation. The resulting model was imported in Pathway Tools in order to create a functional and manually curated PGDB (both PGDBs are available in supplementary materials, the tutorial is available in S1 File). Finally, all the information contained in the model can be exported in a RDF database to be explored using recent computational techniques such as semantic query languages [49–52].

### Wiki-based exploration of metabolic networks: A novel method to explore and monitor GSM reconstructions and their associated metadata

In addition to the easy connection to graph-visualization tools such as Cytoscape [48], PADMet-utils proposes several solutions for exploring GSMs such as the creation of pathway-completeness text reports or graphic reports. As a main originality, a local wiki containing all the information related to the model, including its process metadata and links to external online databases (See Supp. Fig A in S1 File) can be created. For the sake of traceability, we favoured the use of assisted manual curation forms to perform GSM updates. Therefore, the wiki cannot be edited. Its main advantage is to provide a multi-page browsable exploration of thousands of heterogeneous entities related to a GSM together with a semantic search module. This convenient and user-friendly view based on linked data allows to concentrate and trace all the information of the GSM components and the reasons why they were introduced in the network, and also widens it to external information on the web.

**Fig 2:**
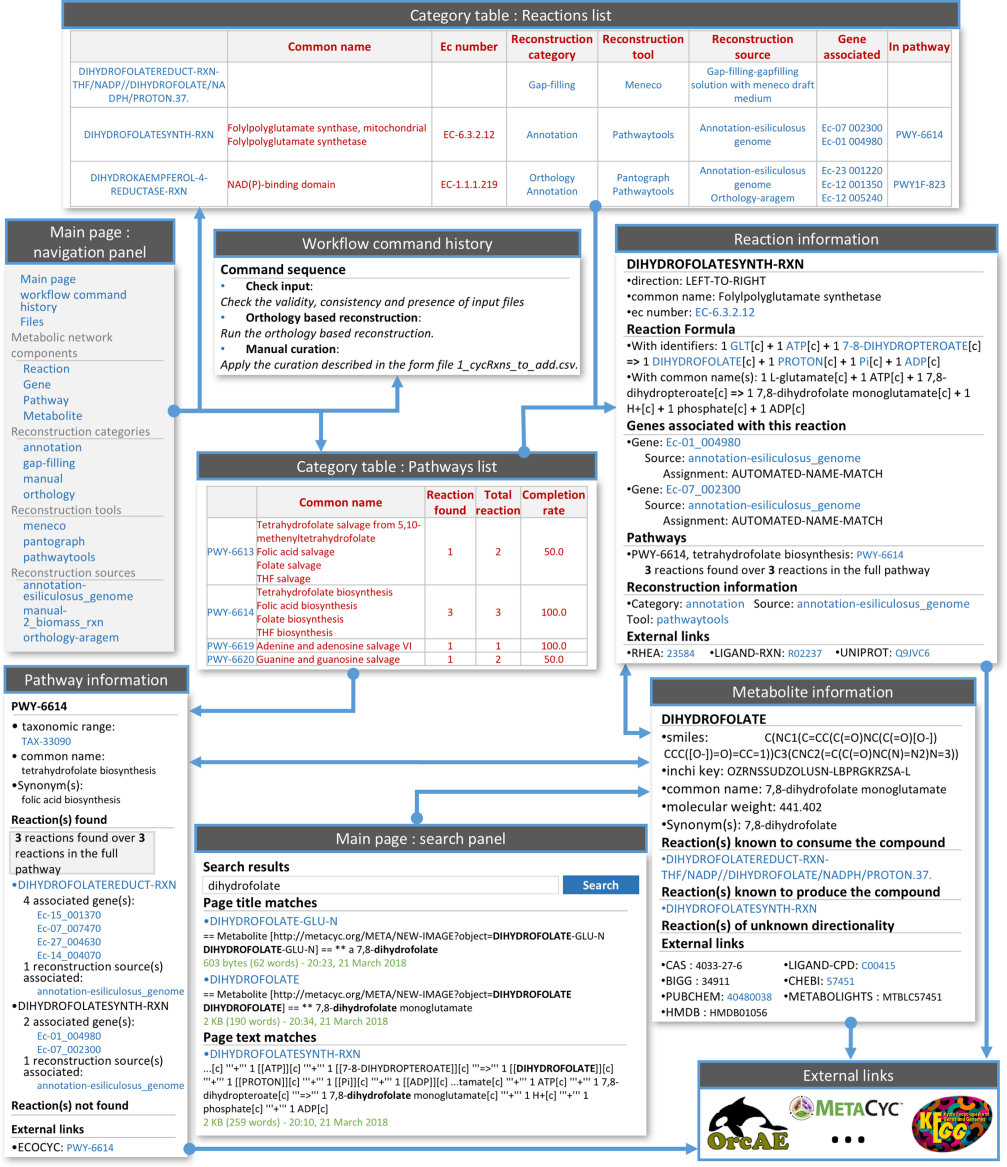
Screen captures of several pages of the local wiki and the interactions between them. A local wiki-based export of the GSM facilitates user-interface exploration and traceability of the reconstruction procedure. Several screenshots of a wiki are displayed, arrows represent the link between pages. Notably, reactions can be sorted and explored according to reconstruction categories, tools and sources. The navigation panel enables exploring and comparing the contributions of each tool used in the “à la carte” GSM reconstruction pipeline. Pathways can be sorted based on their completion rate.

As depicted in Fig 2, the user can browse the contents of the GSM starting either from reactions, genes, metabolites and pathways (when available, which is the case for models relying on Metacyc database) or from the methods used to create the network. In the *E*. *siliculosus* example, the GSM contained 1977 reactions in total: 1661 were recovered from genome annotations, 440 were deduced from orthology-based tools, 85 were added by gap-filling tools and 65 were manually corrected in order to fill biologically-relevant pathways which had a few missing reactions, according to the pathway completeness-rate, after annotation and orthology-based procedures. The wiki can be generated at any step of the pipeline. It is not meant to be edited but automatically re-generated at every step of the reconstruction, for the sake of curation traceability. When the ongoing wiki exploration leads to further insights on the metabolic network reconstruction, such as the need to manually curate the network or use gap-filling tools to complete particular pathways, or the need to include new models for orthology-based completion of the GSM, the user may either decide to integrate a new method in the pipeline or use the assisted manual curation forms to report the corrections. After an update of the model with *AuReMe*, the wiki generation procedure can be run again in order to produce an updated metabolic model which can be further explored with the updated wiki. The wiki exploration is particularly well suited to compare and distinguish the origin of reactions. This is useful to analyse the different components of a metabolic network. For instance, as discussed later, the computation and browsing of orthologs for four different species was a key feature to elucidate the specificities of *T*. *lutea*, for anti-oxidant production.

### Applications of personalized pipelines: Reconstruction of GSMs for four non-model organisms

**Fig 3:**
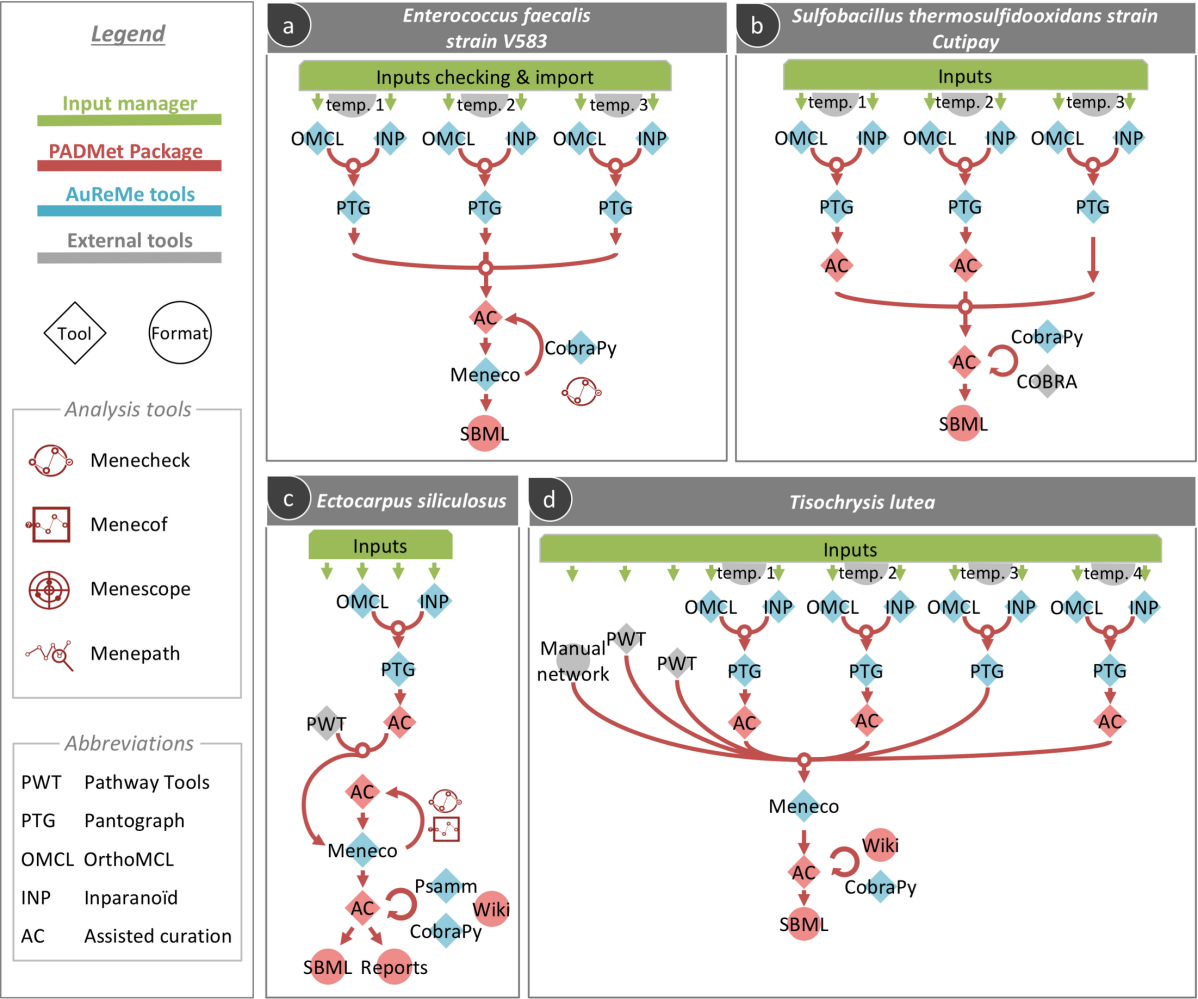
Examples of customizable GSM reconstruction pipelines. *PADMet* allows a user to easily implement flexible and personalized pipelines adapted to the wideness of the considered species and resource data. *PADMet* traces multiple complex reconstruction paradigms. 4 customizations of the reconstruction are presented here: the orthology- and gap-filling-based reconstruction of a) *Enterococcus faecalis* and b) *Sulfobacillus thermosulfidooxidans str*. *Cutipay* models, and the reconstructions of c) *Ectocarpus siliculosus* and d) *Tisochrysis lutea* models, using orthology, annotation and gap-filling. All models include manual curations and several analysis steps.

Here we designed four different pipelines for the genome-scale metabolic reconstruction of a brown alga (*Ectocarpus siliculosus*), a microalga (*Tisochrysis lutea*), and two bacteria (*Sulfobacillus thermosulfidooxidans* strain Cutipay, used in metal extraction processes (biomining) and *Enterococcus faecalis*). GSMs resulting from these pipelines possess the metadata associated with the reconstruction process; enabling the classification of every reaction according to the step that led to its addition in the model, as well as biological metadata. We surveyed all the reconstruction procedures of the four aforementioned organisms to measure the added value from each step of the reconstruction pipelines described in Fig 3. At each step, we gathered information about the model (Supp. Table A in S1 File). Main reconstruction steps for each organism included annotation and/or orthology, merging of models, gap-filling and/or manual curation. Templates for orthology were selected either i) for being the best curated GSMs for organisms that are models in a taxonomic rank of the studied organism, and or ii) for being well-curated models available for taxonomically close organisms. For instance, for *S*. *thermosulfooxidans* str. Cutipay, *Clostridium ljungdahlii* was chosen as a template because it is the phylogenetically closest microorganism in BIGG database. Although *C*. *ljungdahlii* is the closest microorganism, it is anaerobic. As *S*. *thermosulfooxidans* is aerobic, *Bacillus subtilis* was chosen as a representative for aerobic microorganisms and also because of its high-quality published model. *S*. *thermosulfooxidans* and *B*. *subtilis* both belong to the phylum Firmicutes. Finally, *Acidothiobacillus ferrooxidans* was also selected as a template model because it contains the best description of iron and sulfur metabolism, which are of interest regarding *S*. *thermosulfooxidans*. Biologically, it can be argued that horizontal transfer can be observed in this kind of microorganisms, so it makes sense to think they could share common reactions regarding iron and sulfur metabolism.

*E*. *siliculosus* input data was annotation-based reconstruction from Pathway Tools and a template model for orthology-based reconstruction (*Arabidopsis thaliana*, [42]). Final manual curation allowed us to account for expert knowledge and remove two useless reactions. 78.5% of the reactions were associated with gene product information.

*E*. *faecalis* and *S*. *thermosulfooxidans* str. Cutipay GSMs were built only using orthology-based reconstruction from three different organisms’ template models (Fig 3A, 3B and 3C). *C*. *ljungdahlii* iHN637 [34], *B*. *subtilis* iYO844 [53] and *C*. *ferrooxidans* iMC507 *[30]* were used for S. thermosulfidooxidans. Their merging enabled the production of most targets but manual curation was needed to complete the model and simulate growth through Flux Balance Analysis (FBA). 73.3% of the reactions were associated with gene product information. *Escherichia coli* str. K-12 substr. MG1655 [54], *Lactobacillus plantarum* WCFS1 [35] and *Bacillus subtilis* subsp. subtilis str.168 [53] were used as templates for *E*. *faecalis*. Half of the targets were producible after performing orthology, and manual curation enabled the completion of the model for growth simulation with FBA.

Finally, *T*. *lutea* GSM was reconstructed with four template models: *Arabidopsis thaliana*, a land-plant model organism in system biology [42], *Synechocystis* sp. PCC 6803, a well-studied cyanobacteria [29], *Ectocarpus siliculosus*, a brown macroalga model organism [41] and *Chlamydomonas reinhardtii*, a well-studied microalga [40], annotation-based reconstruction from Pathway Tools and a manually created core-model (Fig 3D). Gap-filling procedures were undergone both to restore the biomass producibility and to fill several pathways which were missing a few reactions according to their pathway-completeness rate. Manual curation was performed by selecting relevant reactions from a small-scale network of primary metabolism of *T*. *lutea* called primary network, enabling growth simulation through FBA. Special attention was paid to a carotene-related production pathway (PWY-6475), which was initially incomplete due to insufficient genome annotation and could later be filled by manual curation after assessing orthologue-based information, pathway completeness information and biological information provided by external links. This pipeline customization highlights that using all the available sources of data and combining them lowers the need for gap-filling and manual curation.

### Added-value of the pipeline procedure for pathway completion: Case-study on *E*. *siliculosus* tetrahydrofolate biosynthesis

**Fig 4:**
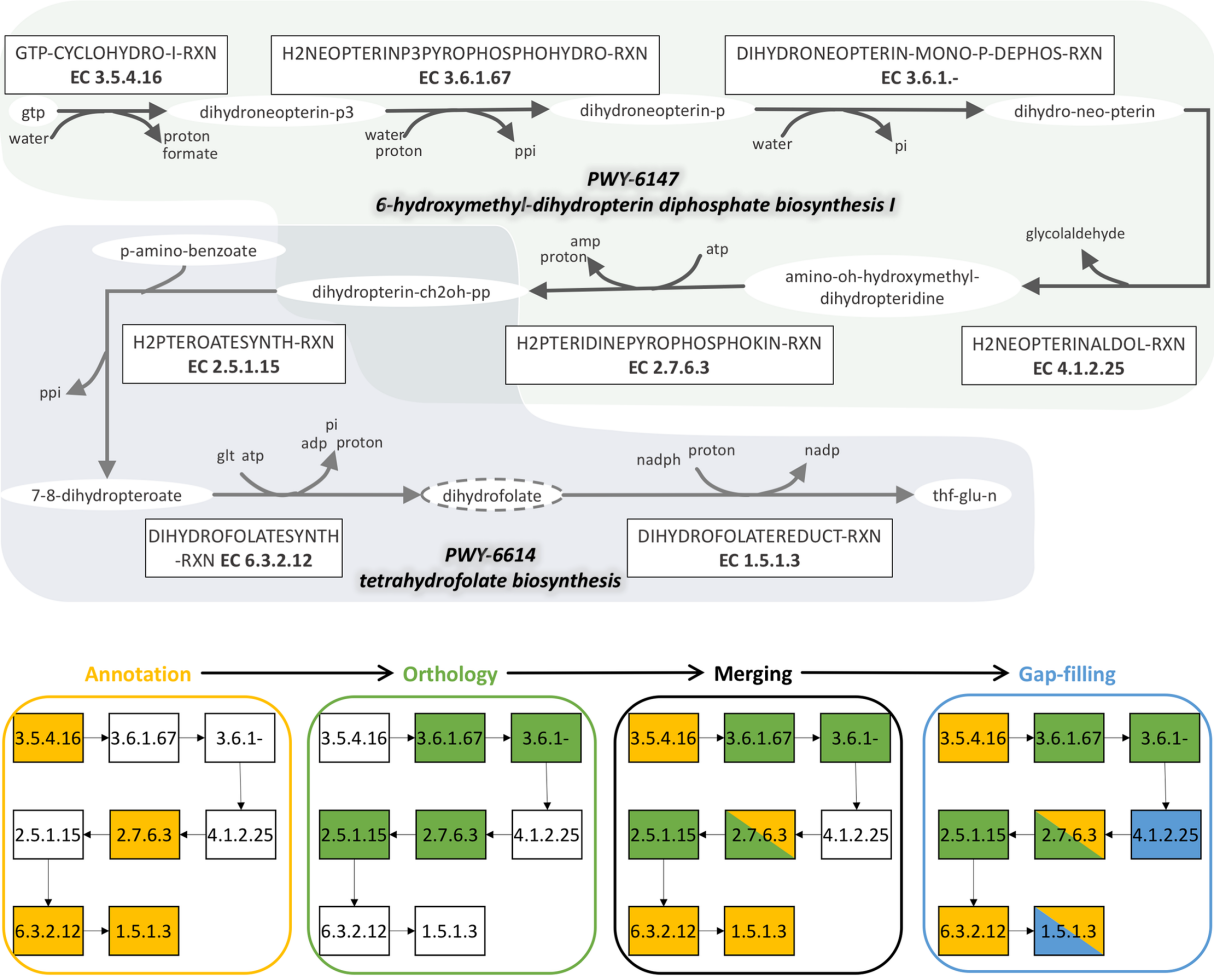
Interest of heterogeneous methods in pathway completion and filling thanks to tracking of process metadata. Completion of the 6-hydroxymethyl-dihydropterin diphosphate biosynthesis I and the tetrahydrofolate biosynthesis pathways in *E*. *siliculosus* via the combination of annotation (yellow), orthology (green) and gap-filling (blue). The dihydrofolate compound with the dotted line is an instance of the dihydrofolate-glu-n class, following MetaCyc classes ontology structure. The class compound is the original reactant of the dihydrofolatereduct-rxn reaction retrieved with annotation, whereas the previous reaction of the pathway (dihydrofolatesynth-rxn) produces the instance dihydrofolate. Hence the gap-filling step that, using an extended version of MetaCyc, selects an instantiated version of dihydrofolatesynth-rxn that consumes the instance dihydrofolate.

The benefit of tracking process metadata during the combination of orthology, annotation and gap-filling is also noticeable at the pathway scale. Complementary methods that exploit all available data can retrieve several reactions from pathways, resulting in the reconstruction of complete or near exhaustive pathways. The wiki page associated with a given pathway describes all the methods of the pipeline providing (multiple)-evidences for the presence of each pathway reaction in the considered species GSMs and allows browsing databases to search for new possible evidences with other species. An example of such pathway completion can be observed during the reconstruction process of *E*. *siliculosus* (Fig 4). Having access to metadata allows the user to check the origin of every added reaction of a pathway.

The 6-hydroxymethyl-dihydropterin diphosphate biosynthesis I (PWY-6147) and the following tetrahydrofolate biosynthesis pathway (PWY-6614) identified in algal metabolism includes in total eight reactions leading to the production of a necessary metabolite, a tetrahydrofolate (THF-GLU-N) starting from GTP. In this example (Fig 4), the need to combine approaches is illustrated by the functional characterization of the pathway after each step of the selected pipeline. Genome-annotation and orthology-based tools identified respectively 3 and 4 reactions of these pathways, including one that was idenfied by both. Combining both information is an essential step leading to a partial reconstruction of the pathways (7/8 reactions) in the merged model.

As mentioned on the MetaCyc website, the database groups reactions and metabolites into classes using an ontology tree structure. In our example, a 7,8 dihydrofolate (DIHYDROFOLATE-GLU-N) is a metabolite class comprising subclasses and instances. The 7,8-dihydrofolate monoglutamate (DIHYDROFOLATE) compound is one of these instances. Originally, reaction DIHYDROFOLATESYNTH-RXN produces DIHYDROFOLATE while the following reaction in the pathway consumes DIHYDROFOLATE-GLU-N. Performing gap-filling with an extended version of the MetaCyc database (provided in the metabolic-reactions.sbml of the database) allows to retrieve an instantiated version of the DIHYDROFOLATEREDUCT-RXN (namely DIHYDROFOLATEREDUCT-RXN-THF/NADP//DIHYDROFOLATE/NADPH/PROTON.37.) that takes DIHYDROFOLATE as a reactant. This enables to restore the producibility of the final compounds of the pathway starting from its inputs. A second reaction is added by gap-filling in PWY-6147. Monitoring with the wiki and the various reports are helpful to keep track of this complex reconstruction process. Application of those heterogeneous methods allows the completion of the entire dihydrofolate biosynthesis pathway from GTP as described in the MetaCyc database.

### Comparing several genomic sources of information to build reliable models and elucidate evolution of metabolic pathways

*T*. *lutea* is a microalga commonly referred to as T-Iso. Recently genomic and transcriptomic investigations were conducted to improve knowledge about this non-model species historically studied due to its use in aquaculture [55]. To obtain a comprehensive overview of this microalgal metabolism, the reconstruction process included metabolic models of the four previously described template organisms. The curation process included gap-filling based on an experimentally built core-network. The analysis of the final model confirmed that a functional T-Iso metabolic network had been obtained despite working with various template models and the related difficulties, especially regarding metabolite and reaction identifier mapping tasks which combined a systematic use of the MetaNetX dictionary [45] and manual curation (Supp. Table A in S1 File).

**Fig 5:**
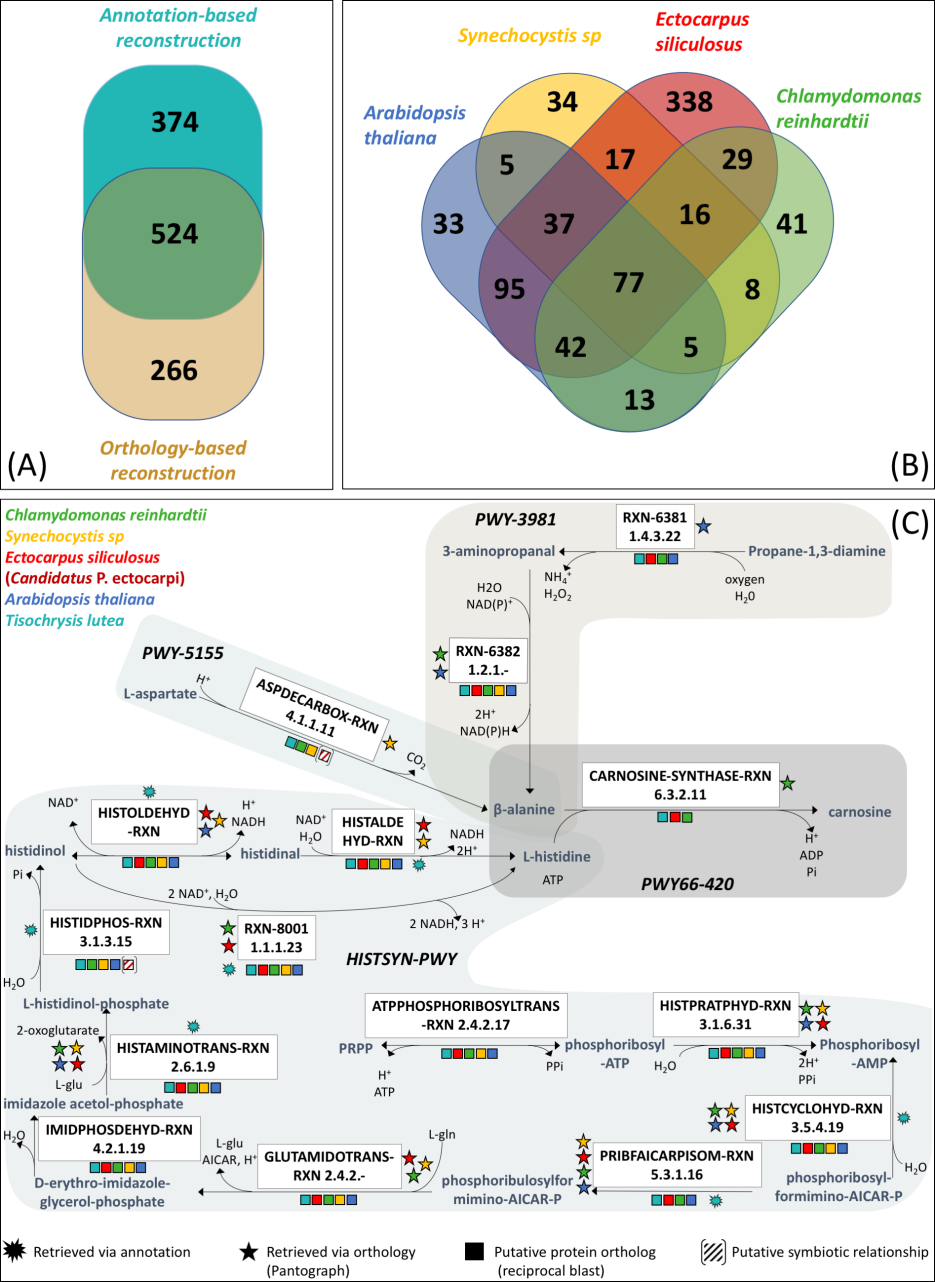
*Tisochrysis lutea* metabolic model exploration: Origin of reactions according to the reconstruction pipeline. (A) Comparison of the numbers of EC numbers introduced in the network either by the annotation pipeline or by the orthology-based analysis 898 enzymes were identified via annotation-based information and 790 enzymes through orthology-based data, among which 524 were already identified via annotation information. (B) *Number of T-Iso ortholog enzymes according to their origin in template models*. For each of the 790 T-Iso ortholog enzymes, the figure depicts in which of the four template models an ortholog of the enzyme had been identified. The four templates used were: *A*. *thaliana*, *C*. *reinhardtii*, *E*. *siliculosus* and *Synechocystis* sp. PCC 6803 to decipher ortholog enzymes in *T*. *lutea*. (C) *T-Iso carnosine biosynthesis*. Reconstruction of T-Iso carnosine synthesis pathway was performed using three sources of data (i) T-Iso genome annotations (cyan star); (ii) template metabolic models (stars) of four organisms: *A*. *thaliana* (blue), *C*. *reinhardtii* (green), *E*. *siliculosus* (red), and *Synechocystis* sp. (yellow) with orthology-based information; (iii) complete proteomes of the four organisms (squares) with sequence alignment information (best reciprocal hit in blasts). All reactions of the T-Iso carnosine biosynthesis are common to the four organisms except for three of them: ASPDECARBOX-RXN, HISTIDPHOS-RXN, and CARNOSINE-SYNTHASE-RXN. The first seems to belong to an alternative pathway to produce β-alanine, also found in *C*. *reinhardtii*, *Synechocystis* sp and *Candidatus Phaeomarinobacter ectocarpi*, a symbiotic bacterium to *E*. *siliculosus*. HISTIDPHOS-RXN was not found in *E*. *siliculosus* but was identified in its symbiotic bacterium *Candidatus* Phaeomarinobacter ectocarpi. CARNOSINE-SYNTHASE-RXN was only identified in algae (*C*. *reinhardtii*, *E*. *siliculosus* and *T*. *lutea*).

Data tracking, ensured by the *PADMet* library and format, allows biological experts to easily identify the origin of specific pathways, reactions and genes in the final metabolic network. Thus, it provides information about the complementarity among the various metabolic network drafts built using genome annotation and orthology-based reconstructions. Considering the 1164 enzymes with an EC-number reported in the network, 374 enzymes come from an annotation-based draft metabolic network only and 266 enzymes are originally associated with at least one of the 4 orthology-based draft metabolic networks (Fig 5A). Contributions of all information are clearly significant. To address issues regarding integrated data origins, pathway completion and their interpretation in biological terms, the *PADMet* representation of the GSM was transposed into a local database in order to be investigated with semantic query languages. Indeed, considering only orthology-based draft networks, it is possible to continue investigations and to associate enzymatic reactions to their metabolic network origin. Fig 5B illustrates the query result: over the 790 enzymes associated with a reaction coming from orthology information, only 77 reactions are associated to the four metabolic network models. 388 reactions originate from *E*. *siliculosus*, reflecting reaction identifiers mapping was facilitated by the use of the same reference database (MetaCyc).

More generally, semantic-based queries and wiki were used to study the specificity of the T-Iso metabolic network. Indeed, the final T-Iso metabolic network led to the identification of a specific antioxidant metabolite: carnosine. In mammals, due to its antioxidant action, carnosine is an essential compound preventing brain neurodegeneration. Recent work about bioactive compounds identification in macroalgae [56] indicate the presence of carnosine in various seaweed species. Thus, this characterization in T-Iso metabolism is of interest for future biological experiments. In the T-Iso GSM, this antioxidant identification was made possible by identification of ortholog proteins between T-Iso and *C*. *reinhardtii* (Fig 5C). Carnosine is a dipeptide consisting of beta-alanine and L-histidine. To complete carnosine production analysis, beta-alanine and L-histidine biosynthesis pathways were carefully examined (Fig 5C). Two complete T-Iso beta-alanine biosynthesis pathways were characterized (PWY-5155 and PWY-3981). Indeed, T-Iso metabolism seems to include an aspartate decarboxylase as a first way to produce beta-alanine (PWY-5155). This enzyme was only identified by orthology (Pantograph) with *Synechocystis* sp. PCC, but based on reciprocal blasts, an ortholog could be existing in *C*. *reinhardtii*. The second beta-alanine biosynthesis pathway (PWY-3981) includes two other enzymes, a diamine oxidase and an aminopropionaldehyde dehydrogenase. This pathway has been characterized in various photosynthetic organisms. So far, T-Iso L-histidine biosynthesis involves a single pathway (HISTSYN-PWY) composed of 10 reactions. L-histidine production pathway identification is confirmed for 8 out of 10 reactions, by genome annotations and/or Pantograph protein ortholog detection with our four template organisms. For the 2 other reactions, ATP-phosphoribosyl transferase (EC:2.4.2.17) and imidazoleglycerolphosphate dehydratase (EC:4.2.1.19), putative ortholog proteins were identified *a posteriori* by a full ortholog protein screening (reciprocal blasts). This analysis suggests that T-iso has the same capability as *C*. *reinhardtii* to produce carnosine, with two production pathways of the precursor beta-alanine. On the contrary, *E*. *siliculosus* should be able to produce beta-alanine with a single production pathway. Interestingly, the pathway involved in its synthesis from aspartate was identified in an obligatory symbiont *of E*. *siliculosus* algal wall: *Candidatus* Phaeomarinobacter ectocarpi [23], whereas the PWY-3981 pathway was not evidenced in this brown alga to date [57]. Ortholog detection of carnosine synthase regarding our various template organisms, also led to the identification of a protein potentially associated to this function in *E*. *siliculosus*. This example illustrates how using several metabolic network models compensates for issues with reaction and metabolite identifiers mapping or missing gene-protein-reaction (GPR) information even in template GSMs. It also enables the integration of expert annotations (i.e. T-Iso core manual network) and information related to phylogenetically close organisms (e.g. *C*. *reinhardtii*) in order to understand better the specificities of a targeted organism (T-iso) with respect to other organisms.

## Discussion

### Customizing, tracing, and exploring GSM reconstruction

Quality GSMs are true knowledge bases integrating both genomic and experimental data of studied organisms. They allow a global picture of an organism’s metabolism, predict growth phenotypes and guide their study. The added value inherent to a GSM is lost if its reconstruction cannot be reproduced or if the information it contains cannot be fully accessed. Unfortunately, this is the case for many of the GSMs that have been generated to date [58]. This problem arises in part because the reconstruction of genome-scale metabolic networks cannot be made without the use of many different tools and databases required to extract the most information possible from available data. Moreover, manual curation steps are also required to generate good quality models. Unfortunately, there is usually no standardized record or metadata related to these steps or of how and when different tools are used in the reconstruction process making it hard to track and reproduce.

We introduce here *AuReMe*, a workspace dedicated to the generation of GSMs that offers solutions to the aforementioned concerns related to their transparency, traceability, reproducibility and exploration [17]. The main objective of *AuReMe* is to keep track of all metadata generated during the reconstruction of the GSM, either metadata linked to the model or its reconstruction process. Reconstruction can be done with high flexibility, both in terms of the database (MetaCyc, BiGG, Model SEED) and the tools/pipelines used for reconstruction. Special attention was given to potential manual curations performed in the network. Manual curations are usually the main metadata to be lost when sharing a model; thus, we simplified and traced the curations via the creation of forms to manually modify the model.

*AuReMe* workspace relies on three levels. i) The database representing the model is the core, as it gathers all data and metadata and is the cornerstone to every application of tool, analysis or modification to the model. ii) The wiki for the visual exploration of the model is a new way to explore large-scale data at multiple levels. iii) The ability to explore more deeply and query the model using RDF standards enables acute analyses to answer biological questions.

Altogether, *AuReMe* offers solutions for GSM reconstruction made to suit user expectations in a world of data in which an emphasis is placed on sharing, tracing and exploration.

### Positioning in the galaxy of GSM reconstruction platforms

The structure of metadata associated to GSM reconstruction can be viewed as a combination of the best practices of two GSM analysis platforms. First, the *PADMet* format can be viewed as an extension of the content of the data files produced by the Pathway Tools platform[13]. In addition to the information related to genome annotation, our approach enables a user to incorporate in the data format any additional information provided by orthology-based, functional gap-filling or curation steps. The traceability and flexibility of the reconstruction procedure is also close to the concept of narratives introduced in the Kbase platform. The *AuReMe* approach shows more flexibility in terms of references since a user can rely on any metabolic network knowledge repository, in contrast to the Kbase platform which exclusively relies on TheSeed database [10].

Similarly, the narratives of the Kbase platform enable the partial investigation of metabolic network attributes (reactions, compounds) through the availability of internet links to The Seed environment databases. In addition, some information about the methods used to assert the presence of a reaction in the network are provided in structured tables which are very close to the structure of the *AuReMe* wiki. The added values of the *AuReMe* technology are threefold. First, it enables the exploration of the full content of the metabolic network metadata either on a local computer or on a shared webserver. Second, the *PADMet* library makes it possible to enrich the wiki with any additional information introduced as an attribute to the *PADMet* format. In particular, when using the MetaCyc database, *AuReMe* enables the exploration of each pathway according to missing reactions, which facilitates the network curation. Thirdly, the capability of exporting the metadata information into a RDF triplestore is in the same line as the semantic analysis modules of the BioCyc database [8]. The main advantage is still increased flexibility since the triplestore can be designed according to user interests. For instance, the metabolic networks for several related species can be stored in the same triplestore in order to facilitate the comparison of their networks with ad-hoc SPARQL requests. The triplestore can also be enriched either with external linked open data (e.g. KOG annotation for enzymes according to their EC numbers, from the KEGG database) or with new data (e.g. expression data in response to several stresses) in order to identify the main pathways associated with particular biological phenotypes.

The main weakness of the *AuReMe* workspace, though, is the command-line interface yielded by the Docker technology. Future improvements may include user-friendly workflow interfaces such as those used in Galaxy technology [15] or the Kbase platform.

### Dependencies to knowledge repositories

The main requirement of the *AuReMe* workspace is the need to select a reference database to ensure the compatibility of all the information introduced in the reconstruction process. This implies that used annotation based draft reconstructions as well as the metabolic networks of template organisms used for orthology-based reconstruction have to be preliminarily conciliated with the reference database. This means that all metabolite and reaction identifiers must be those from the reference database. Even though important progress has been made [45,59], to date, the automatic simultaneous use of more than one database is an unresolved challenge and the correct consolidation of the information still requires significant manual effort [60]. This is because the translation of reaction and metabolite identifiers from one database to another is difficult. Metabolites in different databases are not always presented with the same name or even the same chemical formula or charge. Additionally, equivalent reactions in different databases do not always have the same stoichiometry. If this is not treated carefully, the result can be artefacts in the reconstructed metabolic networks that lead to unrealistic representations of the studied organisms. Moreover, since the universe of reactions and metabolites is large and not fully explored, available databases are not completely exhaustive and therefore many GSMs include a large number of manually annotated compounds and reactions. If they are to be exploited as templates in a reconstruction process, adapting them to fit the identifiers used in the reconstruction is unavoidable. Given this scenario, we believe that currently, the use of a previously selected and, if needed, adapted reference database throughout the reconstruction process is the best way to assure its traceability and reproducibility.

### Towards the study of microbial consortium

Now that the era of the omics sciences is well advanced, the new needs for the reconstruction of metabolic models at the genomic level come from wild or unmodeled organisms, and even more so, the study of organisms in metagenomic samples [61]. It is interesting to note that recently metabolic models at the genomic scale for 773 members of the human intestinal microbiota were generated, showing the useful aspects of this type of reconstruction, but also shows that model reconstruction from large scale metagenomics samples can now be addressed [62]. In addition, whether in biotechnology or health sciences, there are more and more applications in which the use of wild organisms or communities of organisms becomes relevant. Examples range from the use of microorganisms consortia in biomining [63], to address climate change [64], to use organisms as cell factories to improve the production of certain metabolites, or to equip cells with the ability to produce new products [65]. *AuReMe* offers a real opportunity to manage projects where we will need to integrate a plethora of pre-constructed metabolic models, databases and bioinformatics tools to meet the new challenge of exploiting the metabolism of a metagenomic community.

## Methods

### Biological models

We considered two extremophile bacteria, a eukaryote brown alga and a eukaryotic microalga. We deliberately selected species that are distantly related to common model organisms and that have not been studied in much detail. In particular, their genome annotations have not been given special attention and therefore they may contain genes of unknown function, which can generate uncertainties in the model reconstruction process. *Ectocarpus siliculosus* is a model brown alga whose GSM was previously reconstructed [41] but for which recent work provided a new annotation [66]. *Enterococcus faecalis* is a bacterium that plays a role in the dairy industry but is also of high interest in medicine as it may display multiple antibiotic resistances [33]. *Sulfobacillus thermosulfidooxidans* strain Cutipay [67] is a bacterium involved in bioleaching processes. Finally *Tisochrysis lutea* is a haptophyta microalga [55] of aquaculture interest in oyster farming and with a strong potential in the biotechnology field (fatty-acids production).

We used the concepts of seeds and targets to differentiate metabolites during the reconstruction steps. We call seed compounds to the set of metabolites that is available to initiate the metabolism, that is the growth medium. They can also be described as boundary compounds. Target compounds, on the other hand are metabolites whose production is supposed to be achieved by the metabolism of the species under study. They can be components of the biomass reactions or other metabolites that could have been identified as metabolic products in experimental studies.

### The *PADMet* library and PADMet-utils

We developed *PADMet*, a Python library designed to manage metabolic networks in an attribute-based format. This format allows all the biological data to be stored relative to the metabolic network but also the metadata relative to the reconstruction workflow as shown in Supp. Fig A in S1 File. The latter also enables a user to analyse, explore and modify the metabolic network.

The PADMet-utils is a suite of scripts based on the previous library to link admissible input data to the customized workflows and the various analysis tools available in the workspace. It contains four main modules for data management, connection to software, manual curation assistance and model exploration/analysis (S1 File). Regarding the latter, the library proposes several tests to assess the quality of the reconstruction: Flux Balance Analysis, Flux Variability analysis (analysis of essential and blocked reactions), percentage of reactions associated to a gene, determination of mass and/or charge unbalanced reactions using Cobrapy [18]. The connection module of PADMet-utils includes tools to generate RDF-triplestores from metabolic models for further SPARQL queries (see S1 File). Additional details relative to the functionalities of PADMet-utils are available in S1 File.

### MeneTools: Qualitative (graph-based) analyses of GSMs

In order to analyse and curate the four networks studied in this paper, we relied on topological studies as a first step of analysis for draft models. By doing so, the GSM is considered a graph representing reaction and metabolite objects, in which the stoichiometry is not taken into account.

To that end, we developed the MeneTools package (MEtabolic NEtwork TOpological toOLS). This package is based on a recursive combinatorial scheme for producibility which was shown to be relevant for gap-filling [57]. The package enables the detection of unproduced target metabolites in the model (menecheck); the computation of the range of metabolites reachable from a given growth medium set of compounds (menescope); the computation of compounds that could unblock the producibility of targets when added to the model (menecof) and the identification of production paths from compounds of interest starting from a set of seeds (menepath). It is available in the *AuReMe* workspace and as a standalone Python package. The tools solve combinatorial problems using Answer Set Programming.

### Embedded technologies in the *AuReMe* workspace

The *AuReMe* workspace embeds existing tools as well as ad-hoc packages developed to facilitate the interaction between tools (*PaDMet*, Menetools). We used the Docker technology (https://docker.com) to encapsulate the *AuReMe* virtual environment as a container which can be run on any platform (MacOS Yosemite or later, Windows 10, Linux). The following software were installed in the *AuReMe* workspace: Blastall (v2.2.17) [68], CobraPy (v0.5.11) [18], Inparanoid (v4.0)[21], Meneco (v1.5.0) [57], OrthoMCL (vmcl-02-063) [20], Pantograph (v0.2) [22] and PSAMM (v0.28) [19]. In addition, the Docker image was developed to generate another Docker image which encapsulates MediaWiki technologies (https://mediawiki.org) in order to produce the representation of the metabolic model through local wiki webpages (see S1 File for further details).

### *AuReMe* environment user interface and customizability

For all reconstructed networks, The GSM reconstruction workflow was described in a configuration file, which handled the reconstruction process by running simple commands (see details in S1 File). A local database is required to standardize the dataflow during the reconstruction process and to feed the gap-filling and curation steps. The databases MetaCyc 20.0 and BiGG 2.3 were used for the reconstruction of the metabolic networks. They are by default included in the current version of the *AuReMe* workspace, together with the Model SEED database. Notice, however, that the user can alter or import his/her own database from any SBML file.

In order to make the workflow compliant with other pre-installed tools (e.g. running FastGapfill from PSAMM instead of the topological Meneco gap-filling; or running OrthoMCL instead of Pantograph), a simple change in the configuration file is required. More generally, the workflow can be made compliant with any other tool by installing it in the Docker image and then adapting the configuration file to include a rule which ensures the connection between its inputs and outputs and the generic workflow.

## Supporting information

S1 File Supplementary material. Additional results, methods and figures.
(PDF)
