# Supplementary material for: Traceability, reproducibility and wiki-exploration for “à-la-carte” reconstructions of genome-scale metabolic models
Source: PLoS Comput Biol. 2018 May 23;14(5):e1006146. doi: 10.1371/journal.pcbi.1006146 (PMC5988327; doi:10.1371/journal.pcbi.1006146)
Supplement: S1 File — Additional results, methods and figures. (PDF) [file pcbi.1006146.s001.pdf]

SUPPLEMENTARY RESULTS

A package and a workspace to personalize and trace GSM reconstruction

Adaptability to various input data and databases. The PADMet package enables users to link admissible input data to the customized workflows and the various analysis tools available in the workspace (Fig. 1). The *AuReMe* workspace is encapsulated in a Docker image with a “toy” dataset along with versions of MetaCyc 20.0, MetaCyc 20.5 and BiGG 2.3 databases. The purpose of the Docker encapsulation is to provide a workspace that can easily be used with the most diverse set of operating systems (Linux, MacOS, Windows). *PADMet* format ensures the interoperability of knowledge, tools and data. Accepted inputs are genome Genbank files (gbk or gbff), protein sequence fasta files (faa), metabolic models Systems Biology Markup Language files (SBML (1)) for studied or template organisms, Pathway/Genome Databases (PGDB) resulting from Pathway Tools workflows and text files for gene expression data, growth media composition, metabolic targets or biomass components (Fig. 1). An individual can also input a whole new database of reactions and metabolites in a tab-separated value format.

Customization of a pre-set pipeline. The *AuReMe* workspace enables the merging of multiple draft models for an organism under study. Fig. 3 depicts several customizations of pipelines as well as the pre-set pipeline available in the workspace. The latter will import the results of an annotation-based reconstruction of Pathway Tools (PathoLogic) (2) and run a single-template orthology-based reconstruction with Pantograph (3) which reports a consensus between both OrthoMCL (4) and Inparanoid (5). Both models are merged with the *PADmet* package prior performing functional analysis. The functional analysis of a reconstructed metabolic network can be performed at the graph-based level with MeneTools (see Methods) or at the stoichiometric-based level with the functionalities of the pre-installed CobraPy (6) and Psamm (7) packages. When required, reactions can be suggested to fill the network and enable functionality of the GSM. Several tools can be exploited, either separately or iteratively. Meneco, a graph-based gap-filling technique (8) allows the topology of the network to be completed; which can be useful when dealing with exotic organisms. The GapFill (9) or the fastGapFill algorithm (10) allow flux-based completions of the model. The last two are implemented in the pre-installed Psamm (7) package.

Wiki-based exploration of metabolic networks: a novel method to explore and monitor GSM reconstructions and their associated metadata.

GSM information can be displayed in the wiki according to its origin: orthology, genome annotation, gap-filling or manual curation.

Wikis powered by AuReMe display the two families of metadata described in the article. Biological metadata supported by wikis encompass i) all initial data related to an imported model (e.g. conservation of original stoichiometry of reactions, gene associations, data from the ’note’ section of SBML model files, etc.), ii) the reasons that led to a curation of the model, i.e., why a reaction/metabolite was added or deleted (this information is stored when the user completes a form to curate the model), iii) the

corresponding identifiers from different databases for most of the reactions and compounds and iv) the traceability of compounds used as seeds (e.g. growth medium compounds) and targets (compounds known to be produced or biomass components) during model simulations (Supp. Fig. A). GSM reconstruction process metadata include i) the source of each reaction and compound (output of a tool/reconstruction step or organism origin of the model for multispecies modeling), ii) the version of the metabolic database used for data standardization, iii) the steps and tools used during the reconstruction process and iv) the manual curation history i.e. all the *a posteriori* modifications made to the model (Supp. Fig. A).

Wiki pages related to genes, reactions, metabolites or pathways contain both static and linked information. Names, synonyms, formulas, etc., are displayed, in accordance with the data of the original reference database (MetaCyc, BiGG) used to reconstruct the GSM. Links to the latter or other databases (UniProt, Orcae, KEGG) enable the user to easily find external additional information about a gene, a protein or another component of the GSM.

SUPPLEMENTARY FIGURES

Supplementary Figure A. Description of metadata associated to the model or its reconstruction and managed by the AuReMe workspace for storage and wiki-visualization. Biological metadata are related to reactions and metabolites, as well as external identifiers to connect information to other databases. Process metadata describes the history of the modifications applied to the model: steps of reconstruction, manual curation history and origin of model components (which tool, at which step, with which parameter or input file, etc.). Process metadata connect the model with the series of tools that was used to produce it.

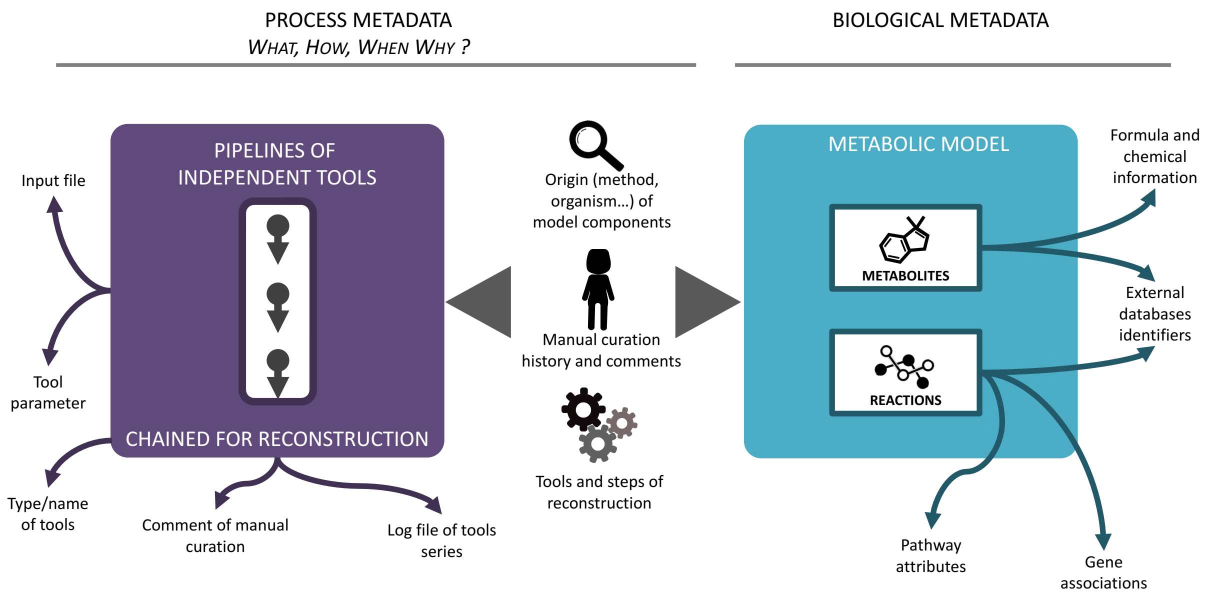

Supplementary Figure B. Abstraction of the RDF semantic-based graph used to represent metabolic models. RDF schema provides data-modeling vocabulary for RDF data. The entities are

linked by relations and may have specific properties (attributes). Some entities used are a cornerstone between two entities to enrich the relation (relation entities). The external metadata entities allow compounds, reactions and pathways to link to external databases such as MetaCyc, BiGG, UniProt and KEGG. The internal metadada entities allow reactions, gene, pathways and metabolic networks to link to information relative either to reconstruction pipeline information or to metabolic network characteristics.

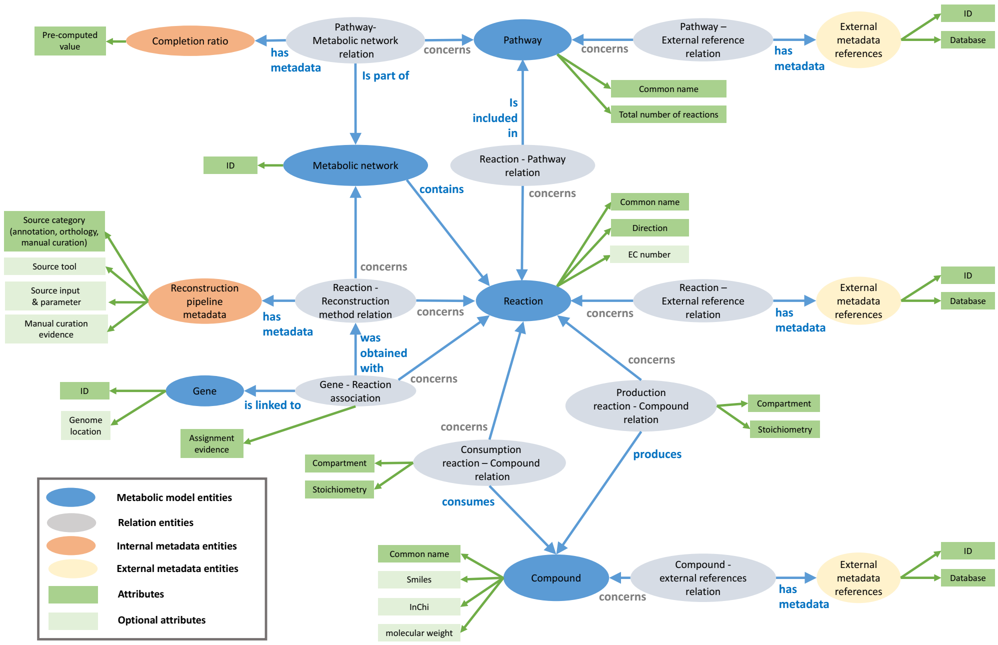

SUPPLEMENTARY TABLE

Supplementary Table A. Impact of the various pipeline steps on the functionality of the built GSM. Network column describes the methodology used at each step of the pipeline. The functionality of each intermediary network was test according to the production of biomass (FBA growth rate), and more generally to the production of a predetermined family of compounds of interest (targets), which contain all the biomass components, with respect to each species.

Organism GSM	Subnetworks of the reconstruction pipeline	number of metabolites	number of reactions	number of genes	ratio of reactions associated to genes	Number of biological compounds of interest (targets)	number of topologically producible targets	number of flux activated targets	FBA growth rate
E. siliculosus	annotation-based network	2118	1834	2281	83%	50	5	5	0
	orthology with *A. thaliana*	650	442	593	89%		1	0	0
	merging annotation and orthology-based models	2118	1887	2281	83%		5	5	0
	**Final network after gap-filling and manual curation**	**2132**	**1977**	**2281**	**79%**		**50**	**50**	**3,02**
Enterococcus faecalis str. v583	orthology with *E. coli* str. K-12 substr. MG1655	728	715	470	100%	92	4	27	0
	orthology with *L. plantarum* WCFS1	472	400	398	100%		4	27	0
	orthology with *B. subtilis subsp. subtilis* str. 168	506	465	374	100%		9	27	0
	merging orthology-based models	937	1192	669	92%		13	37	0
	**Final network after gap-filling and manual curation**	**911**	**1117**	**613**	**84%**		**72**	**92**	**22,54**
T. lutea	experimental annotations	**1108**	**872**	**482**	**75%**	38	**0**	**0**	**0**
	in silico annotations	2088	1696	1855	80%		0	0	0
	orthology with *A. thaliana*	621	366	408	100%		0	0	0
	orthology with *C. reinhardtii*	497	503	408	100%		0	0	0
	orthology with *E. siliculosus*	1590	1139	1163	100%		0	0	0
	orthology with *Synechocystis* sp. PCC 6803	353	207	275	100%		0	0	0
	primary network	285	208	0	0%		0	0	0
	merged models	2743	2779	2728	88%		5	38	74,54
	**Final network after gap-filling and manual curation**	**2749**	**2796**	**2728**	**87%**		**19**	**38**	**74,54**
***S. thermosulfidooxidans* str. Cutipay**	orthology with *Clostridium* iHN637	492	352	297	100%	66	4	3	0
	orthology with *B. subtilis* iYO844	601	507	400	100%		7	3	0
	orthology with *A. ferrooxidans* iMC507	450	309	288	100%		4	3	0
	merging annotation and orthology-based models	831	949	574	91%		23	3	0
	**Final network after manual curation**	**854**	**1067**	**541**	**69%**		**62**	**66**	**6,4**

SUPPLEMENTARY METHODS

AuReMe environment user interface and customizability

For all reconstructed networks, The GSM reconstruction workflow was described in a configuration file (called Makefile), which handled the reconstruction process by running simple commands such as: make orthology-based, make annotation-based, make draft, and make gapfilling. The last two commands run the first two provided they had not been run yet and the corresponding data are available. The configuration file could be personalized in order to select the tools used for each step of the reconstruction workflow. The network analysis was handled through the commands make curation, make menecheck, and make fbacheck. The creation of the wiki pages was handled by make wikipage.

Local and webserver wiki creation

The creation of the wiki for *E. siliculosus* (http://gem-aureme.irisa.fr/ectogem) and *T. lutea* (http://gem-aureme.irisa.fr/tisogem) GSMs was handled in two steps. First, the commands make wikipages in the *AuReMe* workspace launched the creation of the wiki pages for genes, metabolites, pathways and reactions in a local repository of the workspace. Second, the commands make build and make send-all-pages launched the creation of a preconfigured Docker container hosting the wiki infrastructure which could be locally accessed through a web-interface. This local wiki was used to perform the manual exploration and curation of the metabolic reconstructions. Once the networks were curated, the command make web-send-pages uploaded the wiki pages to the webserver on which the Mediawiki technology had been previously installed.

Turning metabolic network information into a RDF triplestore

The script padmet-to-tsv from the module connection of padmet-utils was used to export relations between the entities of the *T. lutea* model that we obtain and the MetaCyc database in padmet format

as TSV files. Based on the RDF graph, shown in Supp. Fig. B, these files were transcribed into RDF triple which were stored into a SPARQL endpoint freely accessible at http://bit.ly/tisoSparql. This representation performed various complicated and precise queries and also made the junction to other databases such as MetaCyc, BiGG, KEGG and UniProt. Based on the latter, SPARQL requests were generated to exhibit pathways which contain exclusive reactions from different sources.

The PADMet library and PADMet-utils

The *PADMet*-utils is a suite of scripts based on *PADMet* library to link admissible input data to the customized workflows and the various analysis tools available in the workspace. The *PADMet*-utils Is contains four main modules for data management, connection to software, data exploration and manual curation assistance. For instance, pgdb-to-padmet from the module connection to software was used to compile the output of Pathway Tools, the PGDB folder to one unique file in *PADMet* format. In the same module, sbml-to-padmet was used to convert one or more SBML to one unique file in *PADMet* format with or without a database of reference. add-seeds-rxn from the module data management was used to add the exchange and transport reactions of a set of metabolites in a given metabolic network. fba-test from the module data exploration was used to perform FBA. To get an idea of the way to use the *PADMet*-utils simply explore the Makefile of *AuReMe*. This toolbox only requires the *PADMet* library and is available in the *AuReMe* workspace or can be downloaded on Gitlab https://gitlab.inria.fr/maite/padmet-utils and used in stand-alone mode.

Exporting a model produced in the workspace to Pathway Tools

AuReMe can be used as an intermediary between the creation of of model in a major platform such as Pathway Tools and its further publication. This enables individual tools to be applied to the model to modify or enhance it while monitoring and tracing the processes. *AuReMe* provides exports in several formats, including a SBML that includes the new reactions and that can be imported into Pathway Tools to re-create a PGDB that can later be published with BioCyc.

The reconstruction of *Ectocarpus siliculosus* metabolic network carried out using the *AuReMe* workflow was built using an initial annotation-based reconstruction made on Pathway Tools (ectov2.2cyc 1.0 in esiliculosus_PGDBs available on http://aureme.genouest.org/suppdata.html), a template model for orthology-based reconstruction (from *Arabidopsis thaliana*) and manual curation.

To go back to the PathwayTools framework we exported the set of reactions added in *AuReMe* in an SBML file and we merged them with the original *Ectocarpus siliculosus* PGDB (ectov2.2cyc), thus creating a new PGDB with the improvements made in *AuReMe*.

To do this, we carried out the following steps:

1) In *AureMe*, we exported an SBML file with the added reactions:

- **Padmet-utils script: sbmlGenerator.py**
- **output file: added_AureMe_PadMet.xml**

Artificial reactions added in *AureMe* for FBA purposes (*i. e.,* exchange reactions) were left out as these reactions are usually not included in PGDBs.

2) In Pathway Tools v 21.0 a new PGDB (addedcyc) was created from the SBML file with added reactions (added_AureMe_PadMet.xml). To do this, in the PathwayTools Pathway/Genome Navigator interface (GUI) we performed the following steps:

- **Step 1:** In the *File* menu, we selected the option "*Import -> SBML File…*" :

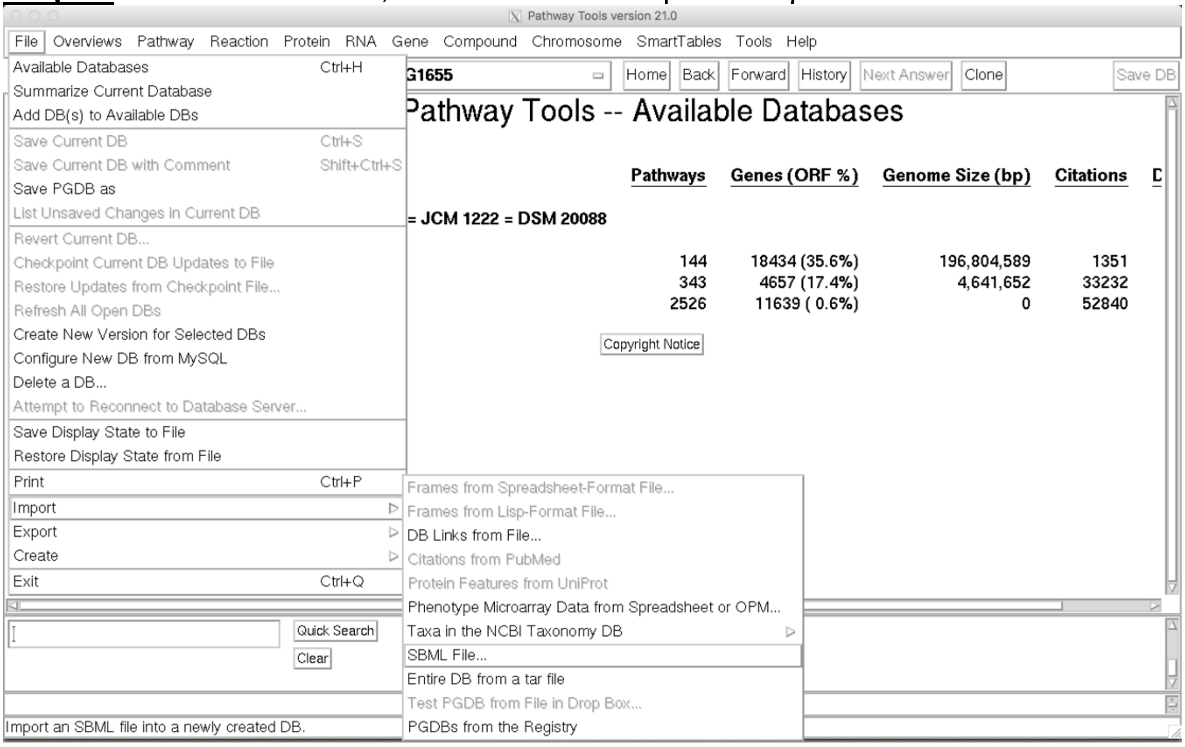

- **Step 2**: In the *SBML import window*, we selected the "*Create a New Database…*" option:

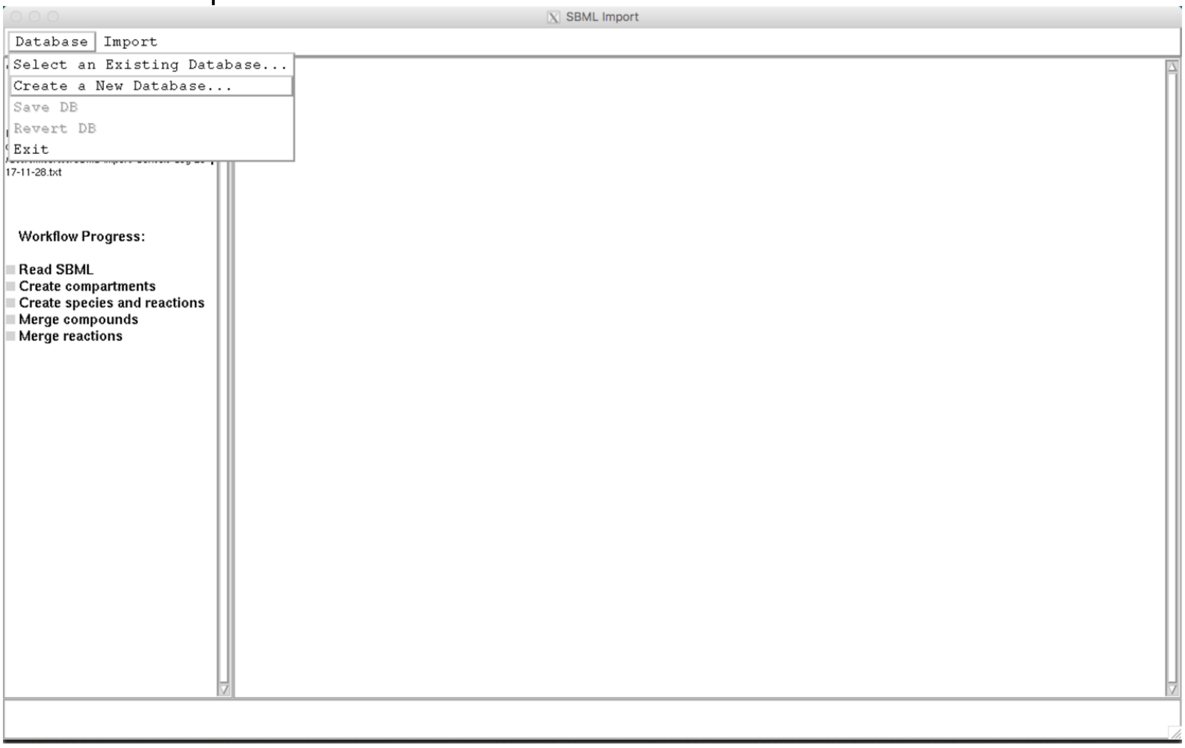

- **Step 3:** In the pop-out window we entered the required information to create our new PGDB (addedcyc):

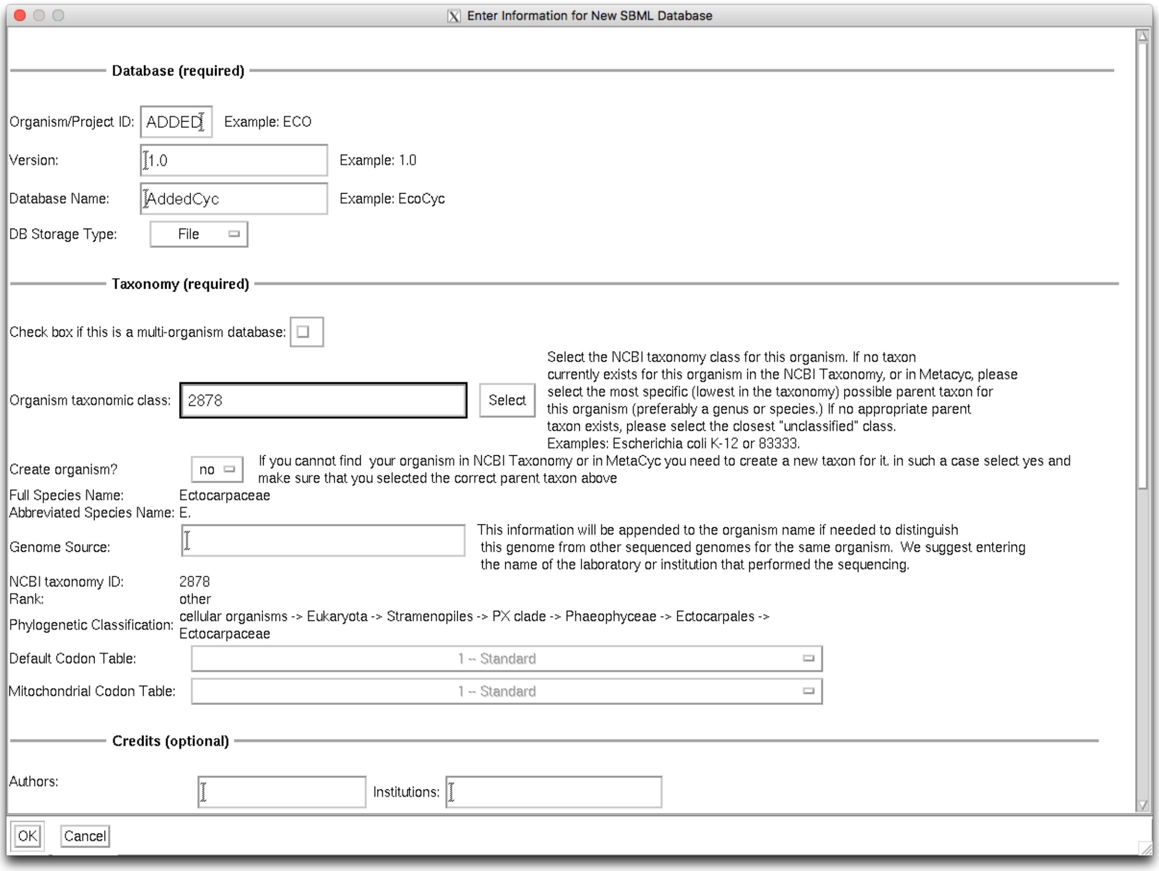

- **Step 4:** Back in the *SBML import window* we selected the "*Import->Select and Read SBML File …*" command and selected our SBML file with the AureMe

added reactions.

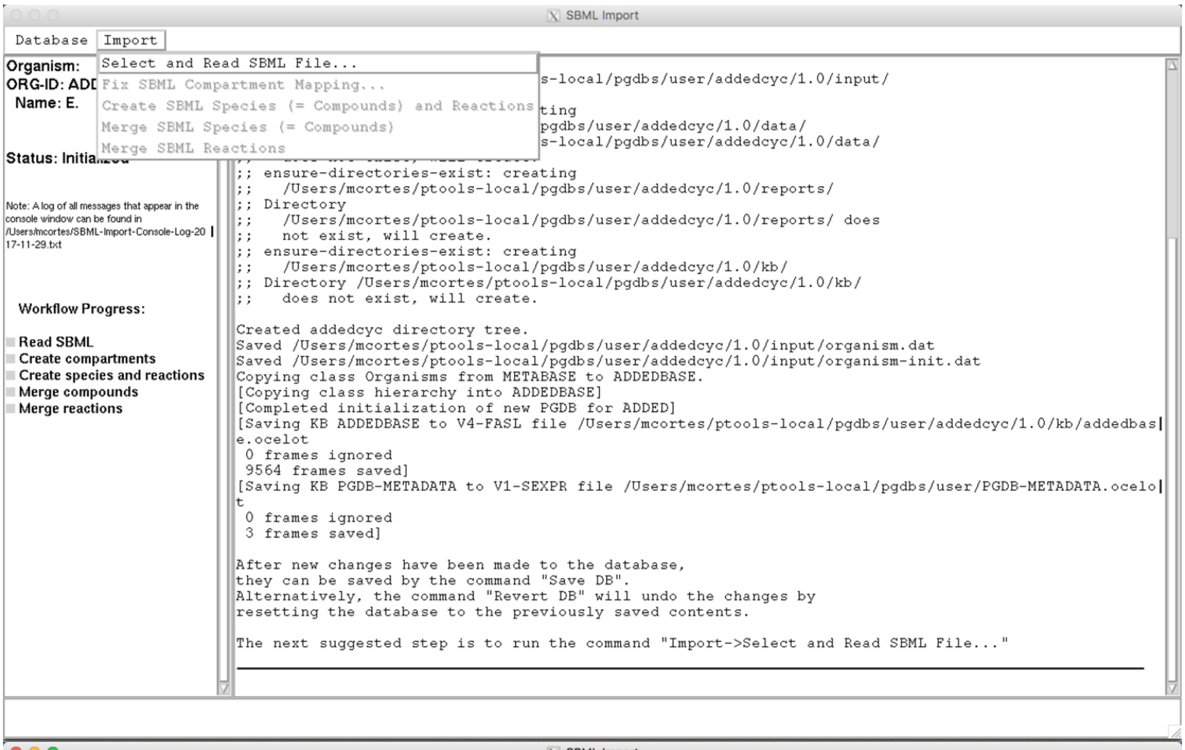

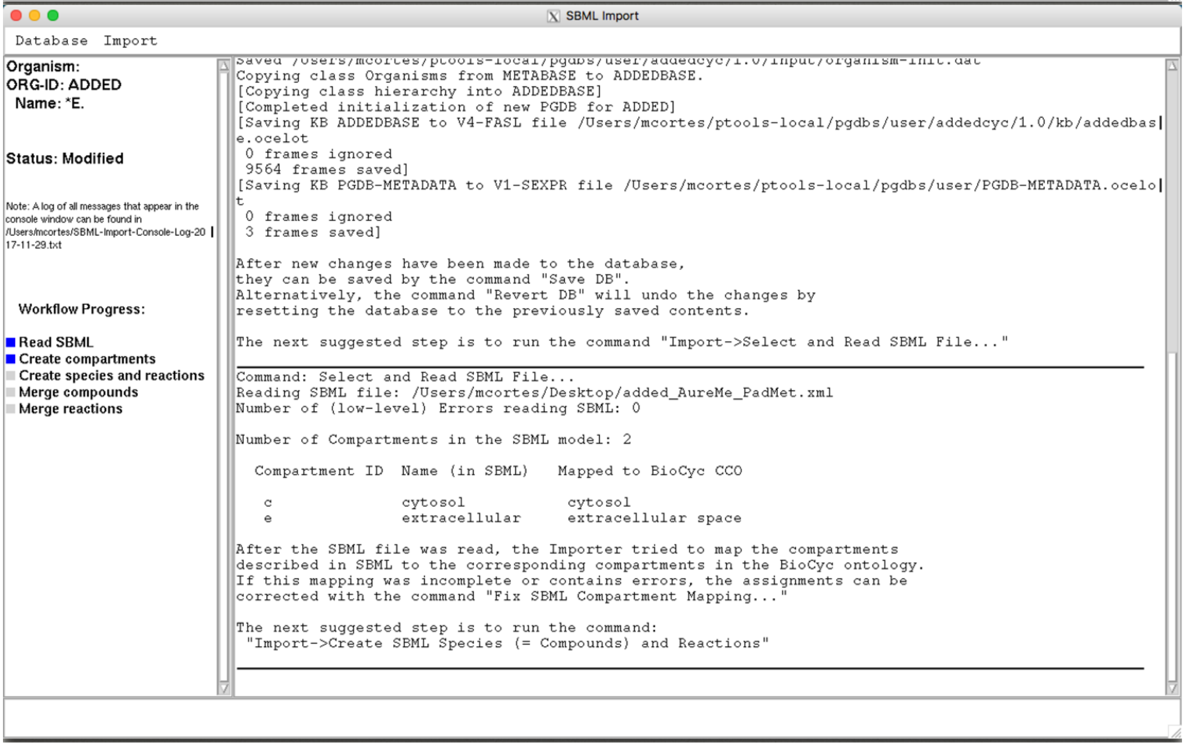

- **Step 5:** We ran the command "*Import->Create SBML Species (i.e. Compounds) and Reactions*". In this step, reactions from the MetaCyc database were

identified.

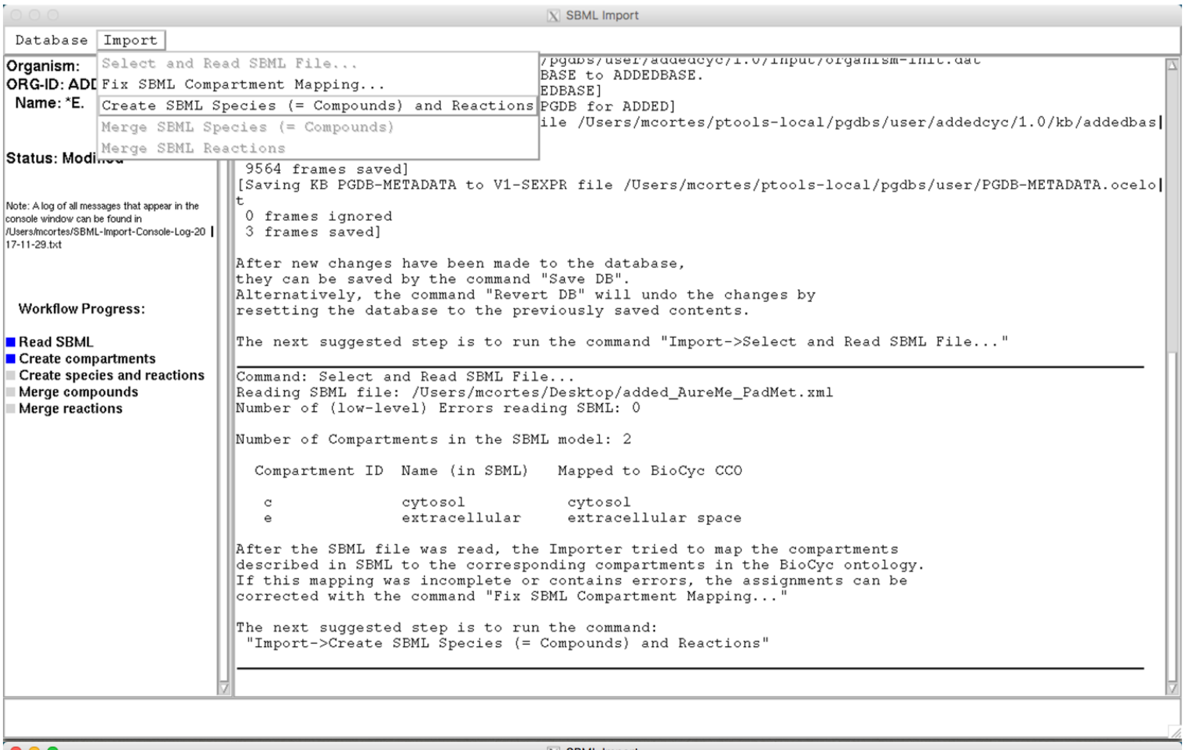

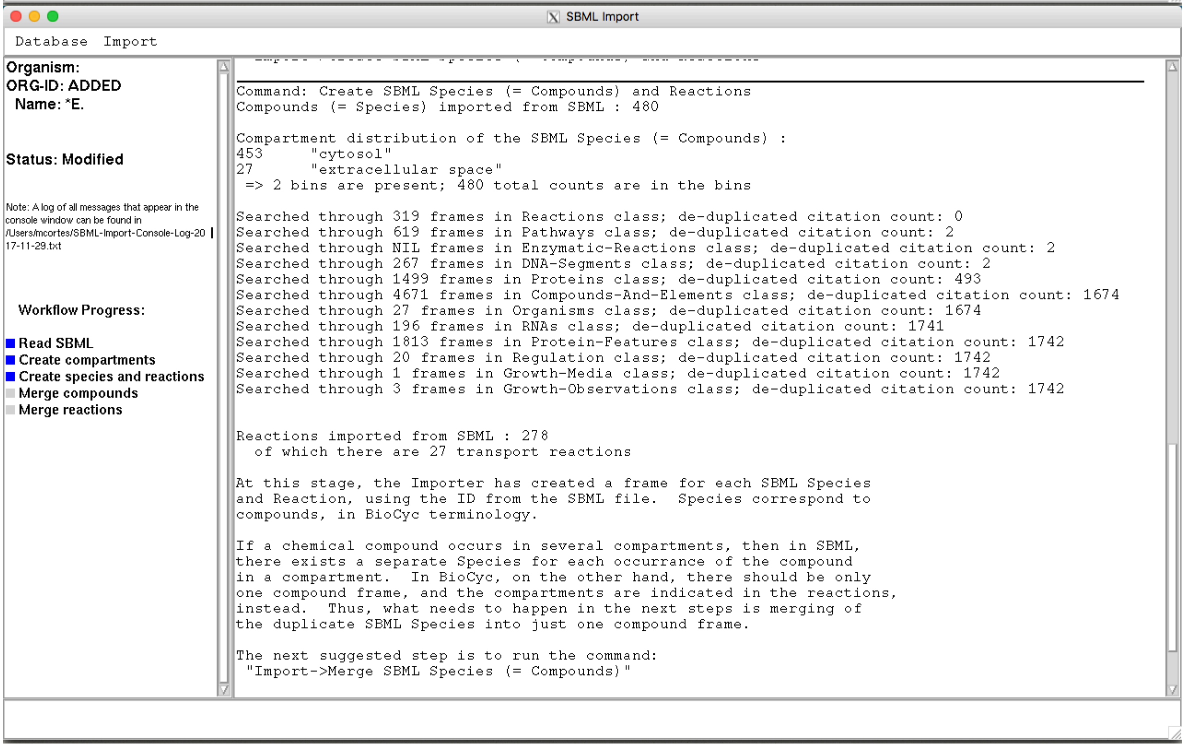

- **<u>Step 6:</u>** We ran the command "*Import->Merge SBML Species (i.e. Compounds)*".

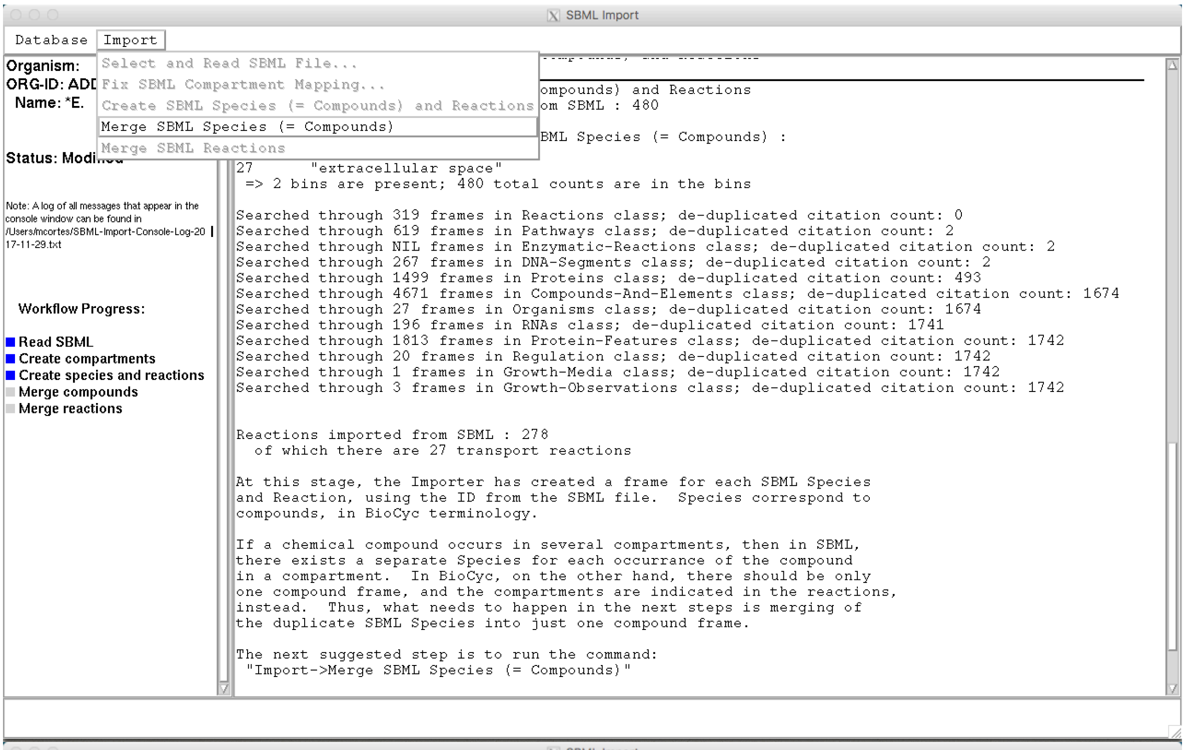
SBML Import
Database Import
Select and Read SBML File...
Fix SBML Compartment Mapping...
Create SBML Species (= Compounds) and Reactions
Merge SBML Species (= Compounds)
Merge SBML Reactions
Organism:
ORG-ID: ADD
Name: *E.
Status: Modi
Note: A log of all messages that appear in the console window can be found in /Users/mcortes/SBML-Import-Console-Log-2017-11-29.txt
Workflow Progress:
Read SBML
Create compartments
Create species and reactions
Merge compounds
Merge reactions
ompounds) and Reactions
om SBML : 480
BML Species (= Compounds) :
27 "extracellular space"
=> 2 bins are present; 480 total counts are in the bins
Searched through 319 frames in Reactions class; de-duplicated citation count: 0
Searched through 619 frames in Pathways class; de-duplicated citation count: 2
Searched through NIL frames in Enzymatic-Reactions class; de-duplicated citation count: 2
Searched through 267 frames in DNA-Segments class; de-duplicated citation count: 2
Searched through 1499 frames in Proteins class; de-duplicated citation count: 493
Searched through 4671 frames in Compounds-And-Elements class; de-duplicated citation count: 1674
Searched through 27 frames in Organisms class; de-duplicated citation count: 1674
Searched through 196 frames in RNAs class; de-duplicated citation count: 1741
Searched through 1813 frames in Protein-Features class; de-duplicated citation count: 1742
Searched through 20 frames in Regulation class; de-duplicated citation count: 1742
Searched through 1 frames in Growth-Media class; de-duplicated citation count: 1742
Searched through 3 frames in Growth-Observations class; de-duplicated citation count: 1742
Reactions imported from SBML : 278
of which there are 27 transport reactions
At this stage, the Importer has created a frame for each SBML Species and Reaction, using the ID from the SBML file. Species correspond to compounds, in BioCyc terminology.
If a chemical compound occurs in several compartments, then in SBML, there exists a separate Species for each occurrance of the compound in a compartment. In BioCyc, on the other hand, there should be only one compound frame, and the compartments are indicated in the reactions, instead. Thus, what needs to happen in the next steps is merging of the duplicate SBML Species into just one compound frame.
The next suggested step is to run the command:
"Import->Merge SBML Species (= Compounds)"

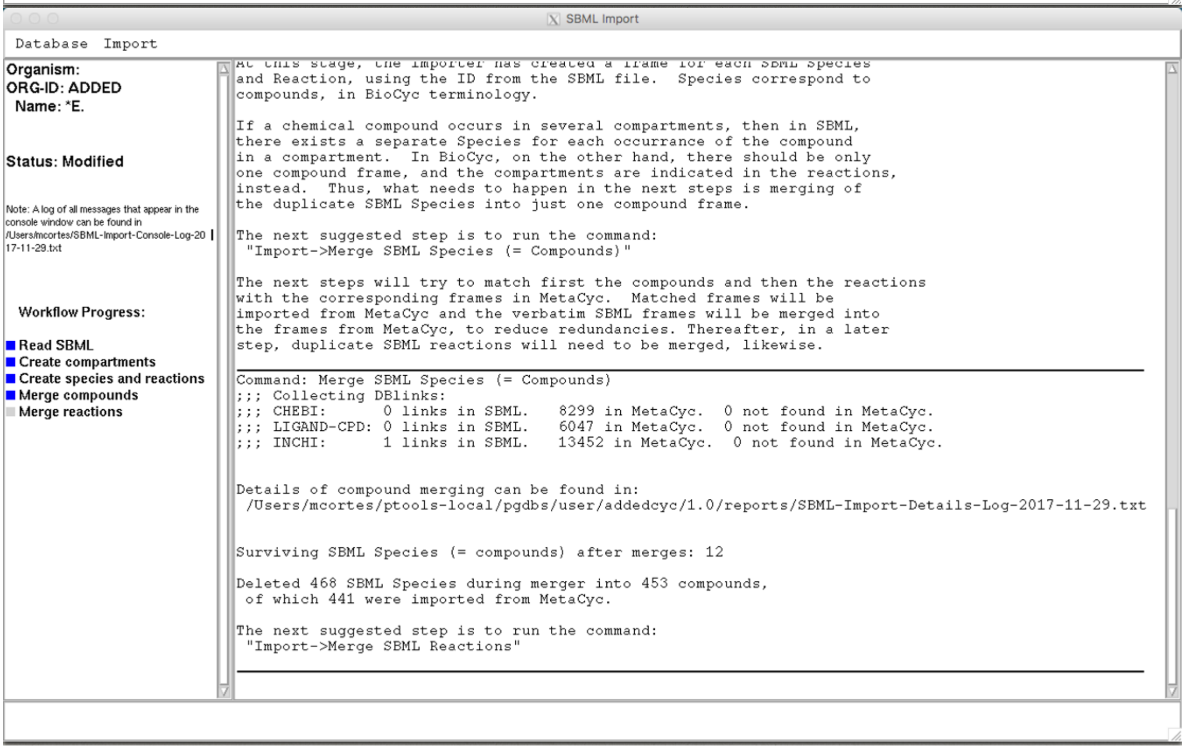
SBML Import
Database Import
Organism:
ORG-ID: ADDED
Name: *E.
Status: Modified
Note: A log of all messages that appear in the console window can be found in /Users/mcortes/SBML-Import-Console-Log-2017-11-29.txt
Workflow Progress:
Read SBML
Create compartments
Create species and reactions
Merge compounds
Merge reactions
and Reaction, using the ID from the SBML file. Species correspond to compounds, in BioCyc terminology.
If a chemical compound occurs in several compartments, then in SBML, there exists a separate Species for each occurrance of the compound in a compartment. In BioCyc, on the other hand, there should be only one compound frame, and the compartments are indicated in the reactions, instead. Thus, what needs to happen in the next steps is merging of the duplicate SBML Species into just one compound frame.
The next suggested step is to run the command:
"Import->Merge SBML Species (= Compounds)"
The next steps will try to match first the compounds and then the reactions with the corresponding frames in MetaCyc. Matched frames will be imported from MetaCyc and the verbatim SBML frames will be merged into the frames from MetaCyc, to reduce redundancies. Thereafter, in a later step, duplicate SBML reactions will need to be merged, likewise.
Command: Merge SBML Species (= Compounds)
;;; Collecting DBlinks:
;;; CHEBI: 0 links in SBML. 8299 in MetaCyc. 0 not found in MetaCyc.
;;; LIGAND-CPD: 0 links in SBML. 6047 in MetaCyc. 0 not found in MetaCyc.
;;; INCHI: 1 links in SBML. 13452 in MetaCyc. 0 not found in MetaCyc.
Details of compound merging can be found in:
/Users/mcortes/ptools-local/pgdbs/user/addedcyc/1.0/reports/SBML-Import-Details-Log-2017-11-29.txt
Surviving SBML Species (= compounds) after merges: 12
Deleted 468 SBML Species during merger into 453 compounds,
of which 441 were imported from MetaCyc.
The next suggested step is to run the command:
"Import->Merge SBML Reactions"

- **<u>Step 7</u>:** We ran the command "*Import->Merge SBML Reactions*".

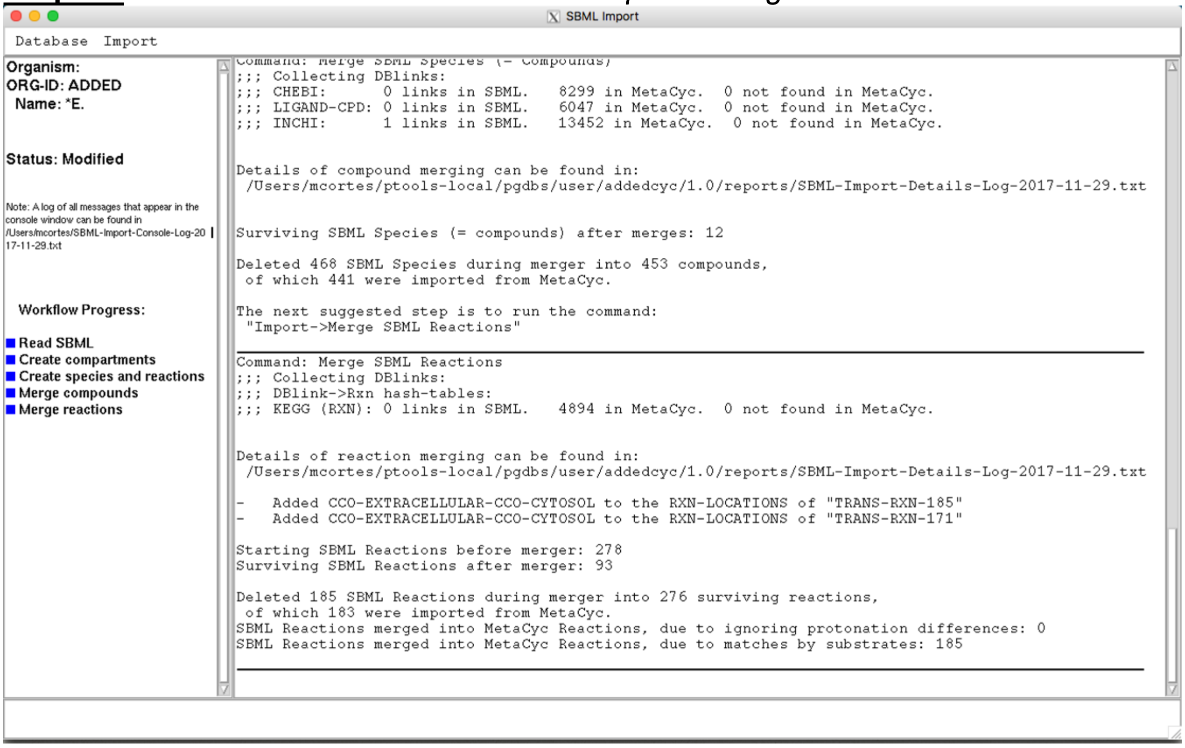

- **<u>Step 8:</u>** Finally, we saved our new **addedcyc** PGDB by running the command *"Database->Save DB*".

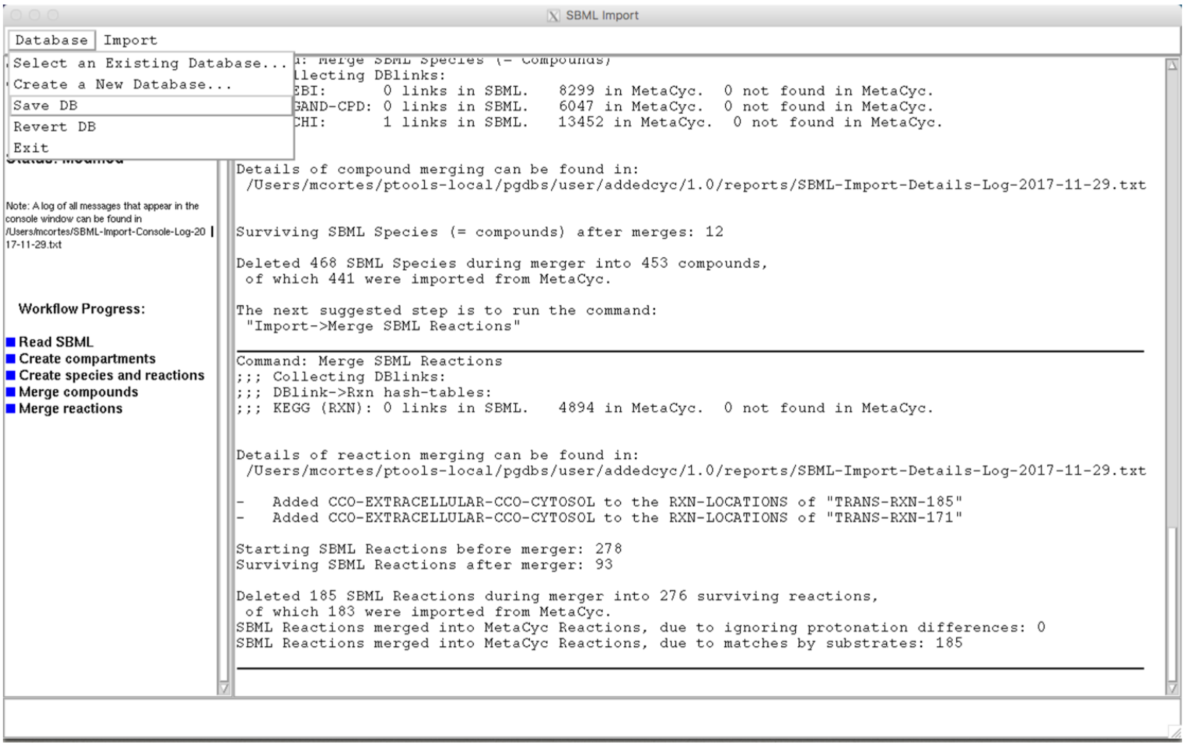

3) In Pathway Tools v 21.0 we exported all reactions in our newly created PGDB (addedyc) to a Lisp-format File. To do this, we started Pathway Tools though the Lisp interpreter with the following command:

- **./Pathway Tools -lisp**

Then, we selected our addedcyc PGDB and exported all reactions to the file "added_frames.txt" with the following commands:

- **(select-organism :org-id 'added)**
- **(export-frames-to-file nil "/PATH-TO-FILE/added_frames.txt" nil :classes '(|Reactions|))**

Where "/PATH-TO-FILE/" is the directory where we stored our frames file added_frames.txt.

4) Then, we selected our original ectocyc2.2 PGDB and we opened the Pathway Tools GUI interface:

- **(select-organism :org-id 'ectov2.2)**
- **(pt)**

5) Finally, we imported the new reactions in "added_frames.txt" into ectocyc2.2 running the command:

- **File->Import->Frames-from-Lisp-format File**

The resulting PGDB is available online: ectov2.2cyc 1.0 in esiliculosus_PGDBs on http://aureme.genouest.org/suppdata.html

Bibliography

1. Hucka,M., Finney,A., Sauro,H.M., Bolouri,H., Doyle,J.C., Kitano,H., Arkin,A.P., Bornstein,B.J., Bray,D., Cornish-Bowden,A., *et al.* (2003) The systems biology markup language (SBML): a medium for representation and exchange of biochemical network models. *Bioinformatics*, **19**, 524–31.

2. Karp,P.D., Paley,S. and Romero,P. (2002) The Pathway Tools software. *Bioinformatics*, **18**, S225--S232.

3. Loira,N., Zhukova,A. and Sherman,D.J. (2015) Pantograph: A template-based method for genome-scale metabolic model reconstruction. *J. Bioinform. Comput. Biol.*, **13**, 1550006.

4. Li,L., Stoeckert,C.J., Roos,D.S. and Roos,D.S. (2003) OrthoMCL: identification of ortholog groups for eukaryotic genomes. *Genome Res.*, **13**, 2178–89.

5. Remm,M., Storm,C.E. V and Sonnhammer,E.L.L. (2001) Automatic clustering of orthologs and in-paralogs from pairwise species comparisons. *J. Mol. Biol.*, **314**, 1041–1052.

6. Ebrahim,A., Lerman,J.A., Palsson,B.O. and Hyduke,D.R. (2013) COBRApy: COnstraints-Based Reconstruction and Analysis for Python. *BMC Syst. Biol.*, **7**, 74.

7. Steffensen,J.L., Dufault-Thompson,K. and Zhang,Y. (2016) PSAMM: A Portable System for the Analysis of Metabolic Models. *PLoS Comput. Biol.*, **12**, e1004732.

8. Prigent,S., Frioux,C., Dittami,S.M., Thiele,S., Larhlimi,A., Collet,G., Gutknecht,F., Got,J., Eveillard,D., Bourdon,J., *et al.* (2017) Meneco, a Topology-Based Gap-Filling Tool Applicable to Degraded Genome-Wide Metabolic Networks. *PLOS Comput. Biol.*, **13**, e1005276.

9. Satish Kumar,V., Dasika,M.S., Maranas,C.D., Wei,J., Goldberg,M., Burland,V., Venkatesan,M., Deng,W., Fournier,G., Mayhew,G., *et al.* (2007) Optimization based automated curation of metabolic reconstructions. *BMC Bioinformatics*, **8**, 212.

10. Thiele,I., Vlassis,N. and Fleming,R.M.T. (2014) fastGapFill: efficient gap filling in metabolic networks. *Bioinformatics*, **30**, 2529–2531.

11. Schellenberger,J., Que,R., Fleming,R.M.T., Thiele,I., Orth,J.D., Feist,A.M., Zielinski,D.C., Bordbar,A., Lewis,N.E., Rahmanian,S., *et al.* (2011) Quantitative prediction of cellular metabolism with constraint-based models: the COBRA Toolbox v2.0. *Nat. Protoc.*, **6**, 1290–307.

12. Agren,R., Liu,L., Shoaie,S., Vongsangnak,W., Nookaew,I. and Nielsen,J. (2013) The RAVEN Toolbox and Its Use for Generating a Genome-scale Metabolic Model for Penicillium chrysogenum. *PLoS Comput. Biol.*, **9**, e1002980.

13. Shannon,P., Markiel,A., Ozier,O., Baliga,N.S., Wang,J.T., Ramage,D., Amin,N., Schwikowski,B. and Ideker,T. (2003) Cytoscape: a software environment for integrated models of biomolecular interaction networks. *Genome Res.*, **13**, 2498–2504.
